# Machine-learning-based spectral methods for partial differential equations

**DOI:** 10.1038/s41598-022-26602-3

**Published:** 2023-01-31

**Authors:** Brek Meuris, Saad Qadeer, Panos Stinis

**Affiliations:** 1 Department of Mechanical Engineering, University of Washington, Seattle, WA 98195 USA; 2 Advanced Computing, Mathematics and Data Division, Pacific Northwest National Laboratory, Richland, WA 99354 USA; 3 Department of Applied Mathematics, University of Washington, Seattle, WA 98195 USA

**Keywords:** Computational science, Applied mathematics

## Abstract

Spectral methods are an important part of scientific computing’s arsenal for solving partial differential equations (PDEs). However, their applicability and effectiveness depend crucially on the choice of basis functions used to expand the solution of a PDE. The last decade has seen the emergence of deep learning as a strong contender in providing efficient representations of complex functions. In the current work, we present an approach for combining deep neural networks with spectral methods to solve PDEs. In particular, we use a deep learning technique known as the Deep Operator Network (DeepONet) to identify candidate functions on which to expand the solution of PDEs. We have devised an approach that uses the candidate functions provided by the DeepONet as a starting point to construct a set of functions that have the following properties: (1) they constitute a basis, (2) they are orthonormal, and (3) they are hierarchical, i.e., akin to Fourier series or orthogonal polynomials. We have exploited the favorable properties of our custom-made basis functions to both study their approximation capability and use them to expand the solution of linear and nonlinear time-dependent PDEs. The proposed approach advances the state of the art and versatility of spectral methods and, more generally, promotes the synergy between traditional scientific computing and machine learning.

## Introduction

In the last 70 years, scientific computing has made tremendous advancements in developing methods for solving partial differential equations (PDEs)^1–3^. Spectral methods constitute a significant part of scientific computing’s arsenal due to their inherent hierarchical structure, connections to approximation theory, and favorable convergence properties^4–7^. Spectral methods generally proceed by expanding the solution of a PDE as a linear combination of basis functions and estimating the coefficients of the linear combination so that the underlying PDE is satisfied in an appropriate sense. Even though spectral methods can be powerful, their effectiveness depends strongly on the choice of basis functions, which is far from obvious for many real-world applications. One source of complications can be the geometry of the domain in which the solution is to be approximated. For example, applications in fluid dynamics often involve complex domains while the frequently used basis functions, e.g., orthogonal polynomials, are suitable only for regular domains^7^. Another source of complications can be the presence of extremely localized features in the solution, e.g., very steep gradients. For example, applications in phase field modeling include the approximation of the order function describing the evolving sharp phase boundary^8^. Due to the global nature of the basis functions used in spectral methods, the resolution of such localized features can decrease the efficiency of a spectral method, unless the particulars of the application are taken into consideration when constructing the basis functions.

In the last decade, due to advancements in algorithmic and computational capacity, machine learning – particularly deep learning – has appeared as a strong contender in providing efficient representations of complex functions^9^. In addition, physics-informed deep learning holds the promise to become a viable approach for the numerical solution of PDEs (see, e.g.,^10,11^). In the current work, we propose a way to combine deep learning and spectral methods to solve PDEs. In particular, we put forth the use of deep learning techniques to identify basis functions to expand the solution of a PDE. These basis functions are custom-made, i.e., they are constructed specifically for a particular PDE and are represented through appropriately defined and trained neural networks.

Our construction starts with candidate functions that are extracted from a recently proposed deep learning technique for approximating the action of generally nonlinear operators, known as the Deep Operator Network (DeepONet)^12^. Due to the intrinsic structure of the DeepONet, the span of these candidate functions is custom-made for a particular PDE (including a class of problem data, e.g., initial/boundary conditions). We have devised an approach to construct a hierarchical orthonormal basis for the candidate space, somewhat akin to Fourier series or orthogonal polynomials, and exploit their favorable properties to expand the solutions of linear and nonlinear time-dependent PDEs. This marks a contrast with methods such as Proper Orthogonal Decomposition^13^ that extract basis functions directly from the data and hence require snapshots of the entire solutions at different time values.

The Universal Approximation Theorem (UAT) of Chen and Chen^14^ guarantees the existence of a pair of two-layer neural networks, termed branch and trunk nets, such that the inner products of their outputs can approximate the action of a continuous nonlinear operator to arbitrary accuracy. This powerful theoretical result was made computationally viable in^12^ by employing deep branch and trunk nets $\{b_k\}_{1 \le k \le w}$ and $\{\gamma _k\}_{1 \le k \le w}$, respectively, and combined via

1 $$\begin{aligned} {{\mathcal {G}}_\text {NN}[\textbf{g}](\textbf{y}) = \sum _{k = 1}^w b_k[\textbf{g}]\gamma _k(\textbf{y})} \end{aligned}$$

Here, $\textbf{y}$ is an evaluation point and $\textbf{g}$ is a vector containing the problem data sampled at a finite number of sensor points (see Methods for more details). The resulting architecture, named DeepONet, enables us to solve PDEs by approximating operators that map the given data (e.g., initial conditions, boundary data, forcing terms, or diffusivity coefficients) to the solutions. Strikingly, the technique is agnostic to the nature of the spatial domain and operates at a much lower computational cost than conventional numerical methods. In addition, complementary error analyses^15,16^ provide upper bounds for the approximation error in terms of network size, operator type, and data regularity, while practical performance demonstrates the low generalization and optimization errors associated with this architecture.

**Figure 1:**
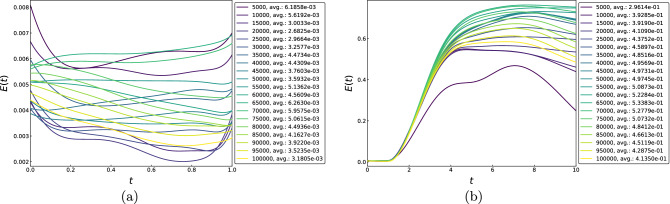
Relative errors for the periodic advection-diffusion problem on $[0,2\pi ]$ using a DeepONet trained for $0 \le t \le 1$, shown for the initial condition $u_0(x) = \sin ^2\left( x/2\right)$, with the number of training epochs going up to $10^5$; time-averaged errors are displayed in the legend. The errors are well under 1% as long as we remain inside the training interval, as seen in (**a**). Extrapolating beyond this interval, however, may lead to large errors, as shown in (**b**) for $0 \le t \le 10$.

Figure 1 shows the results for a DeepONet trained to solve the periodic advection-diffusion problem $u_t + u_x - 0.1u_{xx}= 0$ for $x \in [0,2\pi ]$, applied to the initial condition $u_0(x) = \sin ^2\left( x/2\right)$. The training was performed for $t \in [0,1]$, and the number of epochs increased up to $10^5$. While Fig. 1a shows that the errors are in check for time values in the training domain, the approximate solution quickly loses accuracy outside the training interval, as can be seen in Fig. 1b. This should not be seen as an indictment of the DeepONet approach because it clearly performs satisfactorily on the domain it is designed for. Nevertheless, it leaves room for developing tools that can utilize a trained operator neural network to compute solutions accurately outside the training domain.

In the current work, we present a procedure that harnesses the DeepONet machinery to compute solutions beyond the temporal training interval. Broadly speaking, our approach relies on extracting a hierarchical spatial basis from a trained DeepONet and employing it in a spectral method to solve the PDE of interest (see Methods for further details). By explicitly using the given problem, we expect to be able to generalize beyond the training regime, thus overcoming a limitation associated with small input-output datasets. At the same time, our basis functions inherit the many favorable properties of a trained DeepONet, including excellent representational capability on complex spatial domains and the promise of overcoming the curse of dimensionality. We emphasize that the procedure we propose can, in principle, complement any operator regression technique that can furnish high-quality spatial functions, e.g.,^17–19^. Our technique can also be seen in the context of several important methodologies developed recently combining deep learning methods with variational formulations of PDEs^20–22^.

## Results

In this section, we assess the effectiveness of our approach by applying it to a number of time-dependent problems that possess significantly different qualitative features. For each problem, we take the domain to be $\Omega = [0,2\pi ]$, impose periodic boundary conditions, and denote the initial condition by $u_0$. For the advection-diffusion equation, we also assess our approach for the case of Dirichlet boundary conditions. For each equation, we train a DeepONet to approximate the solution operator that maps $u_0 \mapsto u(t,\cdot )$ for $t \in [0,1]$ (see Sect. 1 in Supplement for details on the training).

**Figure 2:**
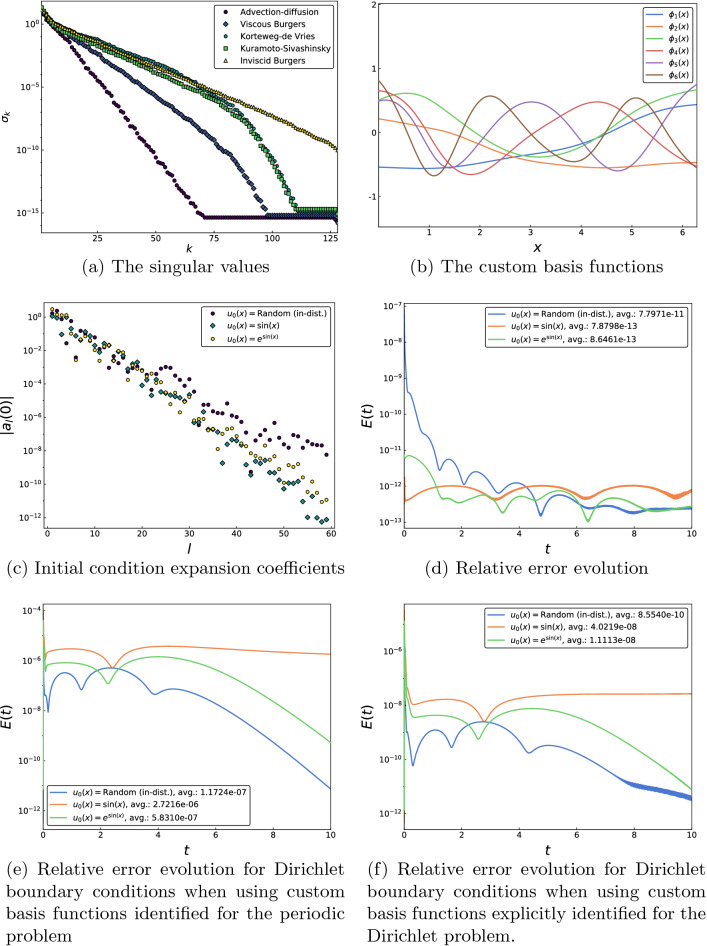
(**a**) The exponentially decaying singular values for all the systems reveal that the basis functions form a hierarchy in terms of their contributions to the trunk net spaces. In addition, the decrease in decay rates for more complex problems suggests that the trunk net spaces for these systems are more descriptive. (**b**) Successive basis functions derived for the advection-diffusion problem (2) can be seen to possess a greater number of zeros and oscillate more frequently. (**c**) The hierarchical structure is also demonstrated by the rapidly decaying expansion coefficients for some smooth example functions, two of which are drawn from outside the training distribution. (**d**) Relative errors from using the custom basis functions to solve the advection-diffusion equation with the initial conditions from (**c**). (**e**) Relative error from using the 59 custom basis functions obtained for the periodic advection-diffusion problem to evolve the advection-diffusion equation with Dirichlet boundary conditions. (**f**) Relative error from using the 73 custom basis functions explicitly identified for the advection-diffusion equation with Dirichlet boundary conditions.

The custom basis functions $\{\phi _k\}$ are extracted from the trunk net function space using a singular value decomposition (SVD) based method. The singular values accompanying each basis function serve as a measure of the contribution of the functions to the trunk net space (see Methods and Sect. 2 in Supplement for more details). We choose a threshold for the singular value magnitude (typically $10^{-13}$ for our numerical experiments) and keep all the basis functions whose corresponding singular values are above the threshold. The rapid decays of the singular values, shown in Fig. 2a for all the systems, are indicative of the hierarchical structure of the basis functions. Moreover, the variation in rates across different problems reflects the intuitive notion that the richness of the trunk net space, measured by its effective dimension, is closely linked with the complexity of the dynamics. As a result, more basis functions are generally allowed for the higher-order problems for the same singular value threshold.

Consider first the advection-diffusion problem

2 $$\begin{aligned} u_t + \alpha u_x - \nu u_{xx} = 0, \end{aligned}$$

with the parameters set at $\alpha = 1$ and $\nu = 0.1$. In Fig. 2b, the first few custom basis functions can clearly be seen to be ordered by increasing oscillatory behavior. An a priori indicator of the suitability of these basis functions for use in a spectral method is the rate of decay of the expansion coefficients $\left\langle \phi _k,u_0 \right\rangle$ for smooth functions $u_0$^4,7^. In Fig. 2c, we assess these rates empirically for a number of smooth functions to be exponential, as for Fourier bases and orthogonal polynomials, suggesting that the custom basis functions are indeed appropriate for use in a spectral procedure. The relative errors from using these example functions as initial conditions to solve (2) using 59 custom basis functions are shown in Fig. 2d. The evolution errors are not only an improvement on Fig. 1a but also decrease rapidly outside the temporal training interval due to an accurate rendering of the diffusion mechanism. Note that these features also hold for the two initial conditions drawn from outside the training distribution. The relative errors from using the 59 custom basis functions identified for the periodic advection-diffusion problem to evolve the advection-diffusion equation with Dirichlet boundary conditions are shown in Fig. 2e. Figure 2f shows the relative errors for the advection-diffusion equation with Dirichlet boundary conditions from using the 73 custom basis functions explicitly identified for the Dirichlet problem.

**Figure 3:**
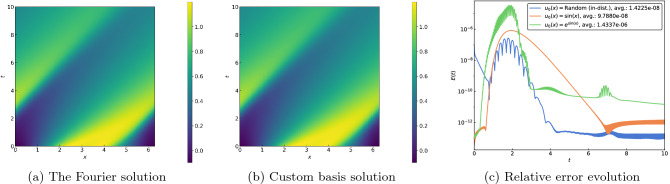
(**a**) Spatiotemporal plot of the solution for the viscous Burgers Eq. (3) using a Fourier expansion for a random in-distribution initial condition. (**b**) Spatiotemporal plot of the solution using the custom basis functions. (**c**) Evolution of relative errors for various initial conditions.

Next, we consider three PDEs that share a common nonlinear term and are distinguished by different regularization mechanisms. These additional terms prevent the formation of corners or discontinuities and lead to notably different qualitative properties. The viscous Burgers equation

3 $$\begin{aligned} u_t + uu_x - \nu u_{xx} = 0, \end{aligned}$$

for example, relies on a diffusive term to smooth over any shocks, with the result that the solution eventually approaches a constant steady state. We set $\nu = 0.1$ and employ 91 basis functions for the results shown in Fig. 3. While the spatiotemporal plots illustrate that our numerical procedure accurately captures the smoothed-out shock and rarefaction waves, the consistently low relative errors in Fig. 3c demonstrate its effectiveness well outside the training regime.

**Figure 4:**
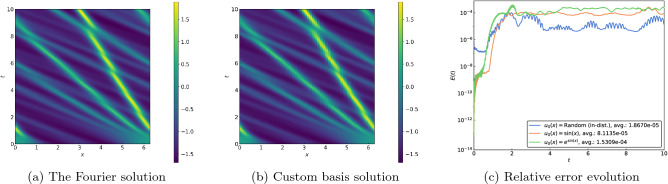
(**a**) Spatiotemporal plot of the solution for the Korteweg–de Vries Eq. (4) using a Fourier expansion for a random in-distribution initial condition. (**b**) Spatiotemporal plot of the solution using the custom basis functions. (**c**) Evolution of relative errors for various initial conditions.

The Korteweg–de Vries equation

4 $$\begin{aligned} u_t + uu_x + \delta ^2 u_{xxx} = 0, \end{aligned}$$

in contrast, employs dispersion to counteract the formation of shocks and famously possesses solutions comprising nonlinearly interacting solitons^23^. Setting $\delta = 0.1$ and using 106 basis functions, we obtain the results shown in Fig. 4. The solitons are represented by the light-colored streaks in the spatiotemporal plots in Fig. 4a and b; their intersections depict the aforementioned nonlinear interactions that are accurately captured by our numerical method. Observe that the errors remain well in check again for time values well beyond the training interval, including for the two initial conditions drawn from outside the training distribution.

**Figure 5:**
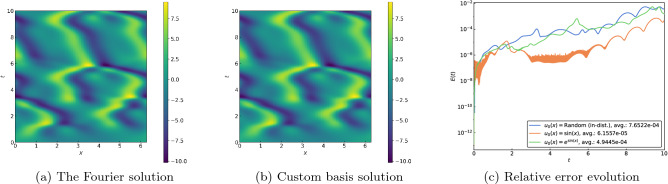
(**a**) Spatiotemporal plot of the solution for the Kuramoto–Sivashinsky Eq. (5) using a Fourier expansion for a random in-distribution initial condition. (**b**) Spatiotemporal plot of the solution using the custom basis functions. (**c**) Evolution of relative errors for various initial conditions.

The Kuramoto–Sivashinsky equation

5 $$\begin{aligned} u_t + uu_x + u_{xx} + \beta u_{xxxx} = 0, \end{aligned}$$

includes a destabilizing anti-diffusion term that is countered by fourth-order dissipation. This system can exhibit chaotic behavior and is a popular model for front propagation^24^. In Fig. 5, we present the results with $\beta = 0.085$ using 105 basis functions for the spectral method. As for the earlier problems, the complicated dynamics are faithfully captured by our spectral method, with the evolution errors kept in control well beyond the training interval and distribution.

Finally, omitting all regularization mechanisms, we end up with the inviscid Burgers equation

6 $$\begin{aligned} u_t + uu_x = 0. \end{aligned}$$

**Figure 6:**
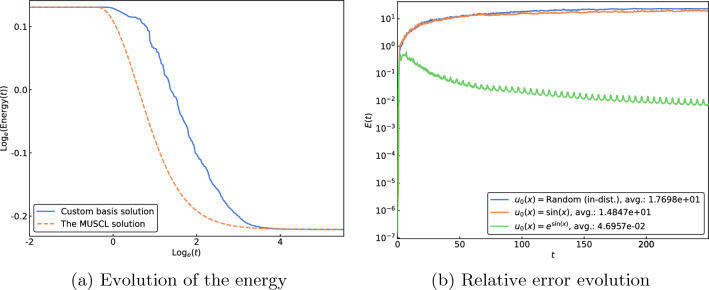
Results for the inviscid Burgers Eq. (6). (**a**) and (**b**) The evolution of the energy depicted on a log-log plot in (a) is obtained for the initial condition $u_0(x) = e^{\sin(x)}$, computed for $t \in [0,250]$. The temporal relative error profiles in (b) are qualitatively similar, with high accuracy while the solutions are smooth giving way to much larger errors on the onset of the shocks, followed by plateauing at the elevated levels.

The solutions of this problem can form shocks in finite time. In the absence of a mechanism to eject the energy that is being consumed by the shock, any spectral approach applied to this problem is prone to large inaccuracies. To accurately capture the evolution of the energy in time, we would need to augment the system with a memory term^25^. This serves to highlight the inherent difficulties of this application and to place the capabilities of the proposed approach without specialized treatment in the larger context of multiscale modeling and model reduction.

## Discussion

We have presented a general framework for using DeepONets to identify spatial functions that can be transformed into a hierarchical orthonormal basis and subsequently used to solve PDEs. We illustrated this framework and its interpolation and extrapolation capabilities by solving five one-dimensional PDEs of varying complexity and exhibiting different qualitative properties. We note that our work should not be construed as an alternative to Fourier methods which possess many favorable properties that make them the optimal choice on periodic domains. Instead, it should be seen as a proof-of-concept that promises to generalize well to complex domains where we do not have classical bases to rely on but can call upon deep learning methods to provide us with candidate basis functions.

The results for the advection-diffusion, viscous Burgers, Korteweg–de Vries, and Kuramoto–Sivashinsky equations with periodic boundary conditions show strong agreement with the Fourier solutions over the entire temporal domain. Additionally, the results for the advection-diffusion equation with Dirichlet boundary conditions show good agreement with the Legendre discontinuous Galerkin solutions over the entire temporal domain when using either the custom basis functions identified for the periodic or the Dirichlet problem (refer to Supplementary Sect. 10 for the results of the advection-diffusion Dirichlet problem trained using non-periodic rather than periodic initial conditions). In particular, the fact that errors remain low for time values well beyond the temporal training interval of the DeepONet demonstrates the temporal extrapolation capabilities of the presented framework. Our approach also performs satisfactorily with initial conditions and parameters different from the training regimes (see Sect. 6 in Supplement for additional results, including for the advection problem). This illustrates the effectiveness of scientific machine learning techniques^26^ because the presented framework consists of embedding the information gleaned from a neural network, which is purely data driven, into the PDE and solving it using conventional techniques.

Results were also presented for the inviscid Burgers equation, which, unlike the other examples whose solutions remain smooth over time when initialized from a smooth initial condition, can develop shocks in finite time. For the time values before the shock, we obtain strong agreement between the custom basis function solution and the ground truth MUSCL solution (see Sect. 6 in Supplement for additional results). However, as evidenced by Fig. 6, around the time instant when the shock forms, the approximate solution becomes more inaccurate and ultimately plateaus at the elevated level of error. This increased level of inaccuracy should not be construed as a shortcoming of the presented framework; instead, this is an issue commonly encountered when using spectral methods for the evolution of singular PDEs^6^. This fact motivated the use of a MUSCL solution to generate the ground truth for training the DeepONets because the use of a Fourier expansion also provides inaccurate results. The inaccuracies occur due to the unavailability of a mechanism to eject the energy that is being consumed by the shock. To account for the ejection of energy and to accurately capture the evolution of the energy in time, we need to augment the system with a memory term (e.g.,^25^). In the case of the inviscid Burgers equation, the inclusion of a memory term allows for energy to be drained from the scales resolved by the simulation^27^. Combining the presented framework with the methods developed in^25^ is an active area of investigation and will appear in a future publication.

For all test PDEs, results were shown for three different initial conditions, one that was randomly selected from within the training distribution and two that were outside the training distribution, $u_0(x) = \sin (x)$ and $u_0(x) = e^{\sin (x)}$. Referencing Figs. 2d–f, 3c, 4c, 5, and 6, strong agreement is shown with the $M=128$ mode Fourier, $L=127$ Legendre polynomial, $M=512$ mode Fourier, or MUSCL solution for all three initial conditions (in advance of the shock in the case of inviscid Burgers). For the viscous Burgers and Korteweg–de Vries equations, we find an increase in the average error over the temporal interval for the out-of-distribution initial conditions compared to the in-distribution initial condition; however, the presented results demonstrate the opportunity to extrapolate not only temporally, but also in terms of the input function space when utilizing the presented framework.

The presented general framework provides many interesting future research directions in addition to those already noted in this section. First, we need to perform meticulous optimization of DeepONet parameters to improve the quality of the custom basis functions. Second is developing a fast custom basis function inverse transform. Preliminary work is underway to develop a fast inverse custom basis function transform using DeepONets. These networks take as the inputs to the branch and trunk nets the expansion coefficients and spatial locations, respectively. Once trained, they will approximate the functions corresponding to the expansion coefficients. In addition, we can train a DeepONet to compute fast the custom basis function forward transform. In particular, we can consider a DeepONet whose trunk net is fixed to output the custom basis functions and the branch net can be trained to output the expansion coefficients. Used together, the forward and inverse transforms will enable the use of a fast pseudo-spectral transform technique so that nonlinear terms can be computed efficiently in real space. Third, as explained in Methods and in Sect. 2 in the Supplement, to preserve the good conditioning of the operations in our construction and enable evaluation away from the quadrature nodes, we perform a final projection of the custom basis functions on Legendre polynomials. As we move to problems on complex domains in higher dimensions, obvious generalizations of Legendre expansions are not available. However, the development of alternative interpolation approaches, based on local spline-based interpolation, partition of unity networks^28,29^, or extension algorithms^30–32^, is an active area of investigation (see the discussion at the end of Sect. 2 in the Supplement). Fourth, the candidate functions (before orthonormalization) were obtained by evaluating the DeepONet trunk net functions at time $t=0$ (see Methods). However, there is nothing precluding the use of candidate functions obtained by evaluating the trunk net functions at times other than $t=0.$ Thus, a more thorough investigation of the time-sampling approach is warranted (see Sect. 7 in the Supplement for preliminary results). Fifth, it is interesting to investigate if the custom-made basis functions developed for one PDE can be used to accurately expand the solution of another PDE (see Sect. 8 in the Supplement for preliminary results). Sixth is a detailed investigation into enforcing the boundary conditions during training using feature expansions and hard constraints^33^. The use of a feature expansion for periodic problems can produce custom basis functions that individually satisfy the boundary conditions so that a purely Galerkin approach may be utilized for evolving the PDEs (see Sect. 9 in the Supplement for preliminary results for the advection equation).

Another interesting avenue for exploration is analyzing the basis functions obtained from DeepONets trained on time-independent problems. Our machinery can be deployed on solution operators for static equations that map, e.g., boundary data, forcing terms, or diffusivity coefficients to the solutions to yield promising custom bases. Eliminating the temporal dimension implies that, along with a possible reduction in the network training cost, the ambiguity associated with using the trunk net functions at $t=0$ as candidate functions would be removed.

We note that the presented framework was initially based off the DeepONet architecture^12^, which is why we explicitly reference the trunk functions; however, there is reason to believe that this framework could be readily extended to other operator neural network architectures, e.g.,^17–19^.

Finally, in the current work we have explored the application of the machine-learning-based spectral methods to partial differential equations that describe prototypical physical mechanisms like advection, diffusion, hyperdiffusion, dispersion and convective nonlinearity with very promising results. Since these mechanisms are prevalent in real-world applications, we are optimistic about the effectiveness of our approach in such settings and is the subject of further investigation.

## Methods

### Architecture of a DeepONet

Let $K_1 \subset {\mathbb {R}}^{d_0}$ and $K_2 \subset {\mathbb {R}}^{d_1}$ be compact, and denote by $C(K_j)$ the space of continuous real-valued functions on $K_j$. Let *V* be a compact subset of $C(K_1)$ and suppose ${\mathcal {G}}: V \rightarrow C(K_2)$ is a continuous, possibly nonlinear, operator. A DeepONet ${\mathcal {G}}_\text {NN}$ is a deep neural architecture designed to approximate ${\mathcal {G}}$^12^. It takes as inputs a discrete representation $\textbf{g} = (g(\textbf{z}_j))_{1 \le j \le m}$ of any $g \in V$, where $\textbf{z}_1,\textbf{z}_2,...,\textbf{z}_m \in K_1$ are pre-selected sensor points, and an output location $\textbf{y} \in K_2$. The DeepONet comprises deep branch and trunk networks $\{b_k\}_{1 \le k \le w}$ and $\{\gamma _k\}_{1 \le k \le w}$, merged together in a dot product layer as in (1):

7 $$\begin{aligned} {{\mathcal {G}}^{\theta }_\text {NN}[\textbf{g}](\textbf{y}) = \sum _{k = 1}^w b_k[\textbf{g}]\gamma _k(\textbf{y})}, \end{aligned}$$

where $\theta$ denotes the trainable parameters. Given input-output function pairs $\left\{ \left( g^{(j)},s^{(j)}\right) \right\} _{1 \le j \le N_f}$, where $s^{(j)} = {\mathcal {G}}[g^{(j)}]$, and corresponding evaluation points $\left\{ \textbf{y}^{(j)}_i \right\} _{1 \le i \le N_p, 1 \le j \le N_f}$, this architecture is trained with respect to the loss function

8 $$\begin{aligned} {{\mathcal {L}}(\theta ) = \frac{1}{N_f N_p}\sum _{j = 1}^{N_f} \sum _{i = 1}^{N_p} \left( s^{(j)}\left( \textbf{y}^{(j)}_i\right) - {\mathcal {G}}^{\theta }_\text {NN}\left[ \textbf{g}^{(j)}\right] \left( \textbf{y}^{(j)}_i\right) \right) ^2.} \end{aligned}$$

### Construction of custom-made basis functions

Let ${\mathcal {G}}$ be the solution operator for a time-dependent problem on spatial domain $\Omega$ that maps the initial condition to the solution at later times. A DeepONet ${\mathcal {G}}_\text {NN}$ of the form (1) is then trained to approximate ${\mathcal {G}}$ with the initial condition $u_0$, sampled at sensor locations $\{\textbf{z}_j\}_{1 \le j \le m} \subset \Omega$, as the input data, and output location $(t,\textbf{x}) \in [0,T] \times \Omega$, where [0, *T*] is the temporal training interval (for more details, see Sects. 1 and 2 in Supplement). We denote the collection of “frozen-in-time” trunk net functions by $\{\tau _k\}_{1 \le k \le p}$, e.g., by evaluating the trunk net functions $\{\gamma _k\}$ at $t=0$ (so that $p = w$, where *w* is the number of trunk net functions used in the DeepONet representation, as in (1)), and normalizing them.

Denote by $\left\langle \cdot ,\cdot \right\rangle$ the $L^2$ inner product on $\Omega$ and let $\{(x_i,\omega _i)\}_{1 \le i \le M}$ be a quadrature rule on $\Omega$ so that $\left\langle h_1,h_2 \right\rangle \approx \sum _{i = 1}^M \overline{h_1(x_i)}h_2(x_i)\omega _i$. The eigenfunctions $\{\phi _k\}_{1 \le k \le p}$ of the covariance operator

9 $$\begin{aligned} {\mathcal {C}} = \sum _{k = 1}^p \tau _k \otimes \tau _k = \sum _{k = 1}^p \tau _k\left\langle \tau _k,\cdot \right\rangle , \end{aligned}$$

ordered by decreasing eigenvalues, form an orthonormal basis for ${\mathcal {S}} = \text {span}\left( \{\tau _k\}_{1 \le k \le p}\right)$ with the following property: for every $r \ge 1$, if we set ${\mathcal {S}}_r = \text {span}\left( \{\phi _k\}_{1 \le k \le r}\right)$, then

10 $$\begin{aligned} \sum _{k = 1}^p \min _{h_k \in {\mathcal {S}}_r} \left\Vert \tau _k - h_k \right\Vert ^2 \le \sum _{k = 1}^p \min _{v_k \in {\mathcal {V}}_r} \left\Vert \tau _k - v_k \right\Vert ^2, \end{aligned}$$

for any *r*-dimensional subspace ${\mathcal {V}}_r$ of ${\mathcal {S}}$. In other words, successive eigenfunctions underpin the optimal lower-dimensional subspaces of the trunk net space, thus making them suitable for use as a custom basis.

Discretizing ${\mathcal {C}}$ and performing its eigendecomposition to compute the basis functions, however, is infeasible in practice because the complexity scales cubically with the size of the quadrature grid. Instead, we define the $M \times p$ matrix *B* by $B_{ik} = \omega _i^{1/2}\tau _k(x_i)$ and perform its SVD $B = QSV^*$. In principle, we can use $$V = \begin{pmatrix} \textbf{v}_1&...&\textbf{v}_p\end{pmatrix}$$ and $S = \text {diag}(\sigma _1,...,\sigma _p)$ to construct

11 $$\begin{aligned} \phi _k = \sigma _k^{-1}\sum _{l = 1}^p \left( \textbf{v}_k\right) _l \tau _l. \end{aligned}$$

However, because this prescription relies on division by singular values that may rapidly decay, the corresponding orthonormal basis calculations can suffer from large errors in practice (see Sect. 2 in the Supplement). Instead, we note that the entries of $W^{-1/2}Q$ provide the values of $\{\phi _k\}$ at the quadrature points via

12 $$\begin{aligned} \phi _k(x_i) = (W^{-1/2}Q)_{ik} \ \text { for } 1\le i \le M \text { and } 1 \le k \le p. \end{aligned}$$

This information about the basis functions needs to be complemented with a suitable procedure to recover their functional forms, enable interpolation away from the quadrature grid, and allow their usage in a spectral method. Of the various alternatives available to us, an orthogonal polynomial expansion is particularly well-suited in the case $\Omega$ is a one-dimensional interval due to our knowledge of the basis functions at Gauss quadrature nodes. For any $L < M$, let $\{q_j\}_{0\le j \le L}$ be the orthonormal Legendre polynomials on $\Omega$ and define the functions $\{\tilde{\phi }_k\}_{1 \le k \le p}$ by

13 $$\begin{aligned} {\tilde{\phi }}_k = \sum _{j = 0}^{L} \left( \sum _{i = 1}^M q_j(x_i)\phi _k(x_i) \omega _i\right) q_j. \end{aligned}$$

This projection enables the evaluation of basis functions away from the quadrature grid. By choosing a sufficiently large *L*, the $\{{\tilde{\phi }}_k\}$ serve as good approximations to $\{\phi _k\}$ while for $L = M-1$, we obtain the exact interpolating polynomials in ${\mathbb {P}}_{M-1}$. More significantly, the procedure only uses (12) and (13), both of which are well-conditioned operations (see Sects. 2 and 3 in the Supplement for more details). We reiterate that our choice of this procedure is motivated primarily by the particular discrete representation of the custom basis functions obtained from (12) and that alternative strategies can also be employed in other settings (see Discussion and Sect. 2 of the Supplement for more details).

The singular values $\{\sigma _k\}$ allow us to gauge the contribution of each basis function to ${\mathcal {S}}$. Once the singular values fall below a certain value, the basis functions are more or less noise and do not contribute significantly to the solution. As a result, we set a threshold, typically $10^{-13}$, and only utilize basis functions corresponding to singular values greater than this cutoff. This leads not only to significant computational savings but also more robust solutions as the noisy functions are weeded out.

### The spectral approach

Without loss of generality, consider a time-dependent partial differential equation

14 $$\begin{aligned} u_t + {\mathcal {N}}[u] = 0, \quad t > 0, \ x \in (b_1,b_2), \end{aligned}$$

with initial condition $u|_{t = 0} = u_0$ and appropriate boundary conditions. Here, ${\mathcal {N}}$ is a (possibly nonlinear) differential operator. Given an orthonormal basis $\{\phi _j\}_{j = 1}^r$, a Galerkin method proceeds by discretizing the solution as $u^r(t,x) = \sum _{j =1}^r a_j(t)\phi _j(x)$ and imposing the constraints

15 $$\begin{aligned} \left\langle \phi _l,u^r_t + {\mathcal {N}}[u^r] \right\rangle = 0, \quad \text {for } 1 \le l \le r. \end{aligned}$$

This yields the system of ordinary differential equations (ODEs)

16 $$\begin{aligned} a_l'(t) = -\left\langle \phi _l,{\mathcal {N}}\left[ \sum _{j = 1}^r a_j(t)\phi _j \right] \right\rangle , \end{aligned}$$

complemented by the initial condition $a_l(0) = \left\langle \phi _l,u_0 \right\rangle$ for $1 \le l \le r$. For our numerical experiments, we have primarily focused on periodic boundary conditions in the interval $[0,2\pi ],$ but this does not limit the applicability of our construction. In the case of periodic boundary conditions, if the basis functions are periodic by construction (e.g., Fourier basis), the boundary conditions are satisfied by default. This is the recipe followed for constructing the ground truth solutions $u_\text {G}$ for the periodic problems used for training and error computation purposes. On the other hand, if the basis functions are not periodic, e.g., the custom basis functions, we simplify (16) further by performing integration by parts and assigning values to the boundary terms that suitably convey information across the interface, as is done for discontinuous Galerkin methods (see Sect. 4 in the Supplement for more details). For all nonlinear examples, the quadratic terms are computed in modal space, while the necessary triple product integrals are pre-computed. The ODE systems of the form (16) are integrated in time using suitable adaptive schemes (see Sect. 5 in the Supplement for additional details). The relative errors in the numerical solution are then computed by

17 $$\begin{aligned} E(t) = \frac{\left\Vert u^r(t,\cdot ) - u_\text {G}(t,\cdot ) \right\Vert }{\left\Vert u_\text {G}(t,\cdot ) \right\Vert }. \end{aligned}$$

## Supplementary Information

Supplementary Information 1.

## Data Availability

The codes used for generation of the data used in this article, along with the development documentation, are available on https://github.com/brekmeuris/DrMZ.jl. The generated datasets are available from the corresponding author on request.

## References

[1] Iserles, A. *A First Course in the Numerical Analysis of Differential Equations*. No. 44 (Cambridge university press, 2009).

[2] Li S, Liu WK (2002). Meshfree and particle methods and their applications. Appl. Mech. Rev..

[3] Tadmor E (2012). A review of numerical methods for nonlinear partial differential equations. Bull. Am. Math. Soc..

[4] Bernardi C, Maday Y (1997). Spectral methods. Handb Numer. Anal..

[5] Boyd JP (2001). Chebyshev and Fourier Spectral Methods.

[6] Hesthaven JS, Gottlieb S, Gottlieb D (2007). Spectral Methods for Time-Dependent Problems.

[7] Canuto C, Hussaini MY, Quarteroni A, Thomas A (2012). Spectral Methods in Fluid Dynamics.

[8] Chen LQ, Shen J (1998). Applications of semi-implicit Fourier-spectral method to phase field equations. Comput. Phys. Commun..

[9] LeCun Y, Bengio Y, Hinton G (2015). Deep learning. Nature.

[10] Karniadakis GE, Kevrekidis IG, Lu L, Perdikaris P, Wang S, Yang L (2021). Physics-informed machine learning. Nat. Rev. Phys..

[11] Alber M, Tepole AB, Cannon WR, De S, Dura-Bernal S, Garikipati K, Karniadakis G, Lytton WW, Perdikaris P, Petzold L (2019). Integrating machine learning and multiscale modeling-perspectives, challenges, and opportunities in the biological, biomedical, and behavioral sciences. NPJ Digit. Med..

[12] Lu L, Jin P, Pang G, Zhang Z, Karniadakis GE (2021). Learning nonlinear operators via DeepONet based on the universal approximation theorem of operators. Nat. Mach. Intell..

[13] Berkooz G, Holmes P, Lumley JL (1993). The proper orthogonal decomposition in the analysis of turbulent flows. Annu. Rev. Fluid Mech..

[14] Chen T, Chen H (1995). Universal approximation to nonlinear operators by neural networks with arbitrary activation functions and its application to dynamical systems. IEEE Trans. Neural Netw..

[15] Deng, B., Shin, Y., Lu, L., Zhang, Z., & Karniadakis G. E. Convergence rate of DeepONets for learning operators arising from advection-diffusion equations. arXiv preprint arXiv:2102.10621 (2021). 10.1016/j.neunet.2022.06.019 35803112

[16] Lanthaler, S., Mishra, S., & Karniadakis, G. E. Error estimates for DeepONets: A deep learning framework in infinite dimensions. arXiv preprint arXiv:2102.09618 (2021).

[17] Li, Z., Kovachki, N., Azizzadenesheli, K., Liu, B., Bhattacharya, K., Stuart, A., & Anandkumar, A. Neural operator: Graph kernel network for partial differential equations. arXiv preprint arXiv:2003.03485 (2020).

[18] Kovachki, N., Lanthaler, S. & Mishra, S. On universal approximation and error bounds for Fourier neural operators. *J. Mach. Learn. Res.*, **22**, Art–No (2021).

[19] Kissas G, Seidman JH, Guilhoto LF, Preciado VM, Pappas GJ, Perdikaris P (2022). Learning operators with coupled attention. J. Mach. Learn. Res..

[20] Ainsworth, M., & Dong, J. Galerkin neural networks: A framework for approximating variational equations with error control. arXiv preprint arXiv:2105.14094 (2021).

[21] Kharazmi E, Zhang Z, Karniadakis GE (2021). hp-vpinns: Variational physics-informed neural networks with domain decomposition. Comput. Methods Appl. Mech. Eng..

[22] Khodayi-Mehr, R. & Zavlanos, M. VarNet: Variational neural networks for the solution of partial differential equations. In *Learning for Dynamics and Control* 298–307. PMLR (2020).

[23] Zabusky NJ, Kruskal MD (1965). Interaction of “solitons” in a collisionless plasma and the recurrence of initial states. Phys. Rev. Lett..

[24] Papageorgiou DT, Smyrlis YS (1991). The route to chaos for the Kuramoto-Sivashinsky equation. Theoret. Comput. Fluid Dyn..

[25] Price J, Meuris B, Shapiro M, Stinis P (2021). Optimal renormalization of multiscale systems. Proc. Natl. Acad. Sci. PNAS.

[26] Baker, N. *et al.**Workshop report on basic research needs for scientific machine learning: Core technologies for artificial intelligence* (Technical report, USDOE Office of Science (SC), Washington, DC (United States), 2019).

[27] Stinis P (2013). Renormalized reduced models for singular PDEs. Commun. Appl. Math. Comput. Sci..

[28] Lee, K., Trask, N. A., Patel, R. G., Gulian, M. A. & Cyr, E. C. Partition of unity networks: Deep hp-approximation. arXiv preprint arXiv:2101.11256 (2021).

[29] Trask, N., Gulian, M., Huang, A., & Lee, K. Probabilistic partition of unity networks: Clustering based deep approximation. arXiv preprint arXiv:2107.03066 (2021).

[30] Boyd JP (2002). A comparison of numerical algorithms for Fourier extension of the first, second, and third kinds. J. Comput. Phys..

[31] Adcock, B. & Huybrechs, D. Approximating smooth, multivariate functions on irregular domains. In *Forum of Mathematics, Sigma*, vol. 8 (Cambridge University Press, Cambridge, 2020).

[32] Matthysen R, Huybrechs D (2018). Function approximation on arbitrary domains using fourier extension frames. SIAM J. Numer. Anal..

[33] Lu, L., Meng, X., Cai, S., Mao, Z., Goswami, S., Zhang, Z., & Karniadakis, G. E. A comprehensive and fair comparison of two neural operators (with practical extensions) based on fair data. arXiv preprint arXiv:2111.05512, (2021).

