# Supplementary material for: Machine-learning-based spectral methods for partial differential equations
Source: Sci Rep. 2023 Jan 31;13:1739. doi: 10.1038/s41598-022-26602-3 (PMC9889394; doi:10.1038/s41598-022-26602-3)
Supplement: Supplementary file 1 — Supplementary Information 1. [file 41598_2022_26602_MOESM1_ESM.pdf]

Supplementary Information

January 11, 2023

1 Generation of ground truth data and DeepONet training parameters

The DeepONets that underlie our construction were trained using the network parameters presented in Supplementary Table 1. All branch layers include bias, and the output layer of the branch network does not apply an activation function. Additionally, the network width was constant for all layers. We sampled $f(\sin^2(x/2))$ from a mean zero Gaussian random field with covariance kernel,

$$\kappa_l(x_1, x_2) = e^{\frac{-\|x_1 - x_2\|^2}{2l^2}}, \quad l = 0.5, \tag{1}$$

to generate the random training and testing initial conditions. Twenty-five example initial conditions are shown in Supplementary Figure 1. The in-distribution initial conditions for each partial differential equation (PDE) are presented in Supplementary Figure 2. The out-of-distribution initial conditions tested for each of the example PDEs are shown in Supplementary Figure 3. To indicate the effects of random initialization of the neural networks, the mean testing error and corresponding standard deviation based on three training runs are presented in Supplementary Table 2 for each example PDE with periodic boundary conditions, and in Supplementary Table 3 for the advection-diffusion equation with Dirichlet boundary conditions.

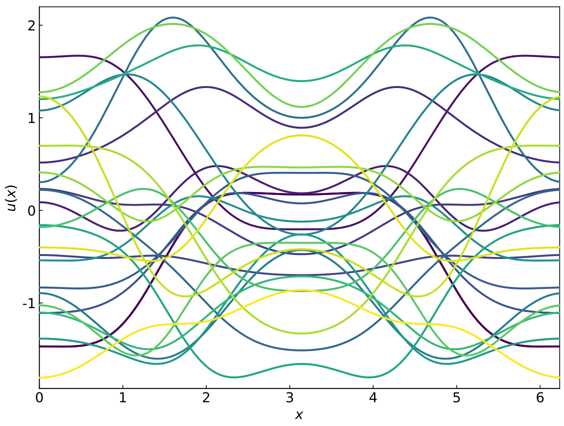

Supplementary Figure 1: Twenty-five example initial conditions sampled from the Gaussian random field.

The ground truth data for the advection, advection-diffusion, and viscous Burgers equations with periodic boundary conditions was generated by writing the solutions in terms of $M = 128$

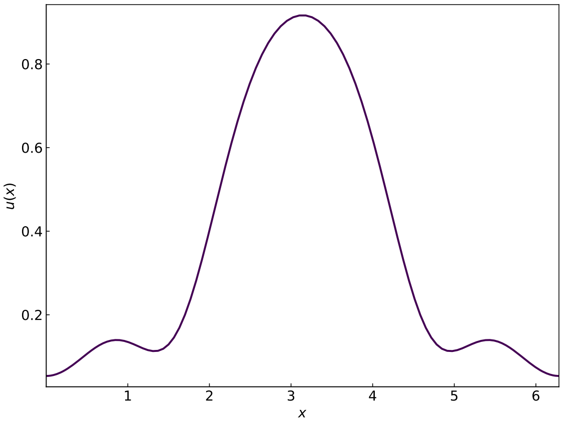

(a) Advection

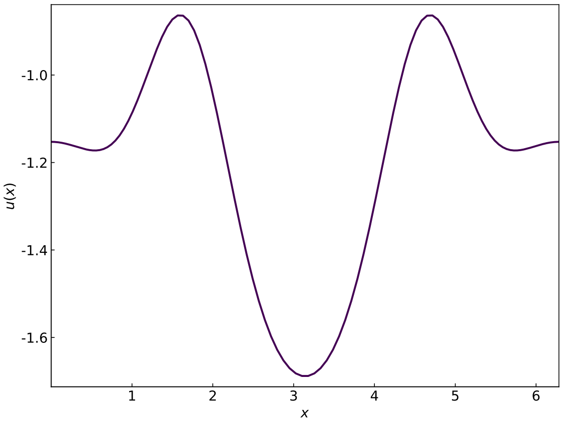

(b) Advection-diffusion

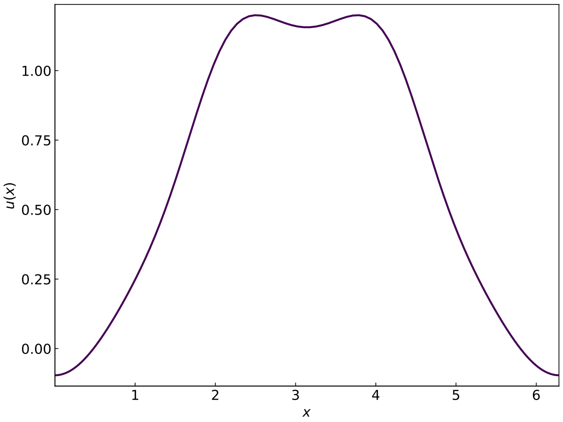

(c) Viscous Burgers

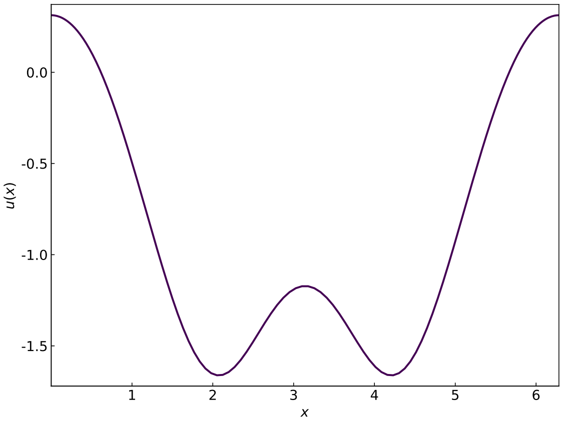

(d) Korteweg–de Vries

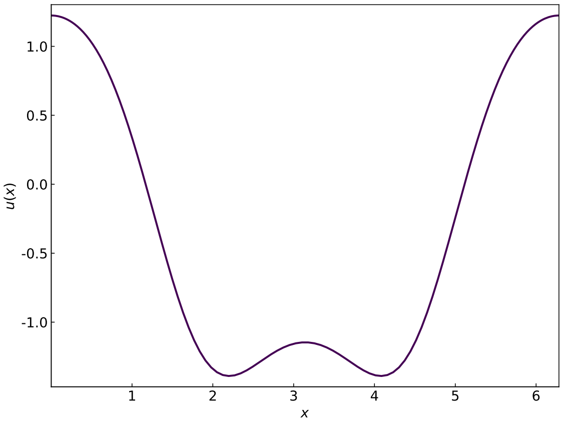

(e) Kuramoto–Sivashinsky

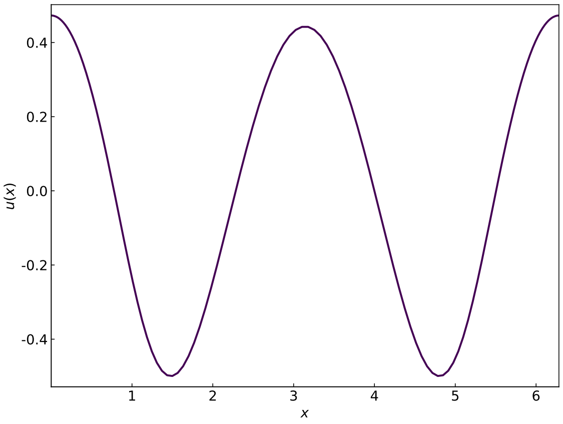

(f) Inviscid Burgers

Supplementary Figure 2: Random in-distribution test initial conditions for each PDE.

Fourier modes,

$$u_{\mathrm{G}}^{M}(t,x) = \sum_{k=-M/2}^{M/2-1} \hat{u}_k(t) e^{ikx}, \tag{2}$$

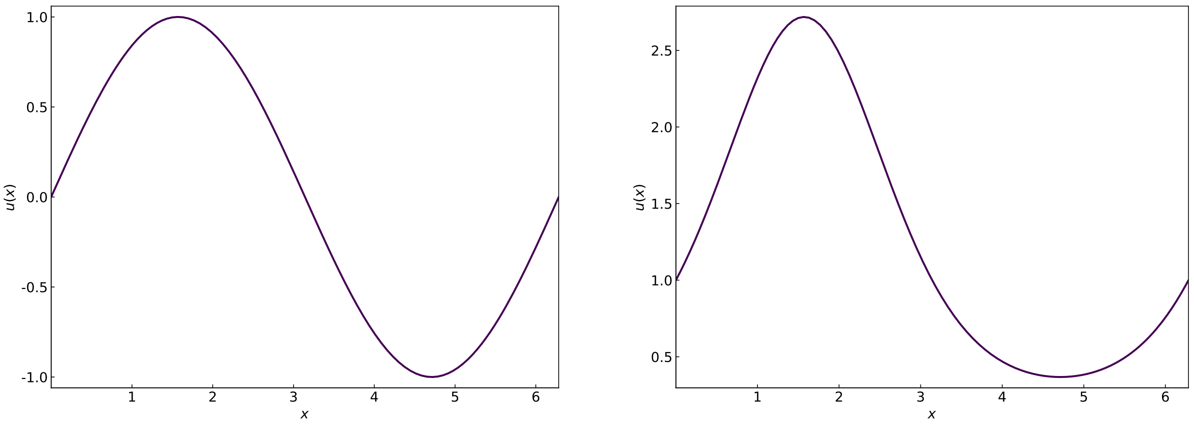

(a) $u(0, x) = \sin(x)$

(b) $u(0, x) = e^{\sin(x)}$

Supplementary Figure 3: Out of distribution test initial conditions, $u(0, x) = \sin(x)$ and $u(0, x) = e^{\sin(x)}$, for each PDE.

with the most negative mode set to zero to avoid asymmetry between the positive and negative modes [1]. The resulting systems of differential equations were solved for $t \in [0, 1]$ using a Runge–Kutta–Dormand–Prince integrator with adaptive step size, relative error tolerance 10^{-10}, and absolute tolerance 10^{-14} [2]. The solution was saved at time values 10^{-3} apart for the linear and 10^{-4} for the nonlinear PDE examples. The convolution sum that results from using (2) for solving the viscous Burgers equation was evaluated by padding the Fourier solution using the 3/2-rule for de-aliasing [1], transforming the solution to real space, and then computing the fast Fourier transform (FFT) of the product of the real space solution with itself.

The ground truth data for the advection-diffusion equation with Dirichlet boundary conditions was generated by writing the solution in terms of $L = 127$ orthonormal Legendre polynomials,

$$u_{\mathrm{G}}^{L}(t, x) = \sum_{j=0}^{L} \hat{u}_j(t) q_j(x), \tag{3}$$

and with the boundary conditions enforced using a discontinuous Galerkin approach. Refer to Supplementary Section 4 for additional details. The Dirichlet boundary conditions were specified to be the values of the initial condition at the endpoints $x = 0$ and $x = 2\pi$ for all time. The resulting system of differential equations was solved on a 128-node Gauss–Legendre quadrature grid for $t \in [0, 1]$ using an explicit, singly diagonal, implicit Runge–Kutta integrator with adaptive step size, relative error tolerance 10^{-10}, and absolute tolerance 10^{-14} [2]. The solution was saved at time values 10^{-3} apart.

The ground truth data for the Korteweg–de Vries and Kuramoto–Sivashinsky equations was generated by writing the solution in terms of $M = 512$ Fourier modes (2), with the most negative mode again set to zero to avoid asymmetry between the positive and negative modes. The 3/2-rule and the pseudo-spectral transform was again utilized to evaluate the resulting convolution sums. The resulting system of differential equations for the Korteweg–de Vries equation was solved for $t \in [0, 1]$ using an explicit, singly diagonal, implicit Runge–Kutta integrator with adaptive step size, relative error tolerance 10^{-8}, and absolute tolerance 10^{-12} [2]. The resulting system of differential equations for the Kuramoto–Sivashinsky equation was solved for $t \in [0, 1]$ using a Crank–Nicolson integrator with adaptive step size, relative error tolerance 10^{-8}, and absolute tolerance 10^{-12} [2]. To

Parameter	Setting
Activation functions	Tanh
Optimizer	Adam
Error	Mean squared error
Learning rate	0.00001
Number of training epochs	50000
Number of sensors	128
Number of solution locations	100
Number of training initial conditions	500
Number of testing initial conditions	1000
Branch net depth	2
Branch net width	128
Trunk net depth	3
Trunk net width	128
Weight initialization	Glorot uniform
Bias initialization	Zero
Mini-batch size	100

Supplementary Table 1: Parameter settings for training the DeepONets.

PDE	Mean	Standard deviation
Advection	3.37×10^{-5}	3.59×10^{-6}
Advection-diffusion	6.90×10^{-5}	2.69×10^{-5}
Viscous Burgers	2.36×10^{-3}	1.79×10^{-4}
Korteweg–de Vries	3.05×10^{-2}	2.95×10^{-5}
Kuramoto–Sivashinsky	6.58×10^{-2}	4.24×10^{-4}
Inviscid Burgers	1.23×10^{-2}	2.44×10^{-4}

Supplementary Table 2: DeepONet mean testing errors and standard deviation for all the example PDEs based on three training runs each.

PDE	Mean	Standard deviation
Advection-diffusion	6.98×10^{-5}	3.23×10^{-5}

Supplementary Table 3: DeepONet mean testing errors and standard deviation for the advection-diffusion equation with Dirichlet boundary conditions based on three training runs.

train the DeepONet, the solution for the Korteweg–de Vries and Kuramoto–Sivashinsky equation was saved 10^{-4} time units apart and down sampled from the 512 solution locations to the 128 uniformly spaced sensor locations.

The ground truth data for the inviscid Burgers equation was generated by utilizing a MUSCL (monotonic upwind scheme for conservation laws) scheme with a second-order Roe scheme for the flux and a minmod slope limiter [3, 4]. The spatial domain was discretized using 4096 points, and the resulting equations were solved for $t \in [0, 1]$ using a Bogacki–Shampine 3/2 integrator with

adaptive step size, relative error tolerance 10^{-6}, and absolute tolerance 10^{-8} [2]. To train the DeepONet, the solution was saved 10^{-4} time units apart and down sampled from the 4096 solution locations to the 128 uniformly spaced sensor locations.

2 Further orthonormalization details

In this section, we provide further details about our procedure for extracting an orthonormal basis from a given trunk net space. In particular, we explain the reasoning underlying our method, explore alternative (but ultimately less successful) approaches, and provide further intuition regarding the various quantities and operations in play.

Denote by $\langle \cdot, \cdot \rangle$ the L^2 inner product on Ω:

$$\langle h_1, h_2 \rangle = \int_\Omega \overline{h_1(x)} h_2(x) \, dx, \tag{4}$$

and let $\{(x_i, \omega_i)\}_{1 \le i \le M}$ be a quadrature rule on Ω such that

$$\langle h_1, h_2 \rangle \approx \langle \mathbf{h}_1, \mathbf{h}_2 \rangle_{(\mathbf{x}, W)} := \mathbf{h}_1^* W \mathbf{h}_2, \tag{5}$$

where $\mathbf{x} = (x_i)_{1 \le i \le M}$, $W = \mathrm{diag}(\omega_1, \dots, \omega_M)$, and $\mathbf{h}_l = (h_l(x_i))_{1 \le i \le M}$ for $l = 1, 2$.

Let $\{\tau_k\}_{1 \le k \le p}$ be a collection of linearly independent functions on Ω that have been normalized (i.e., $\|\tau_k\| = 1$ for all k). Set $\mathcal{S} = \mathrm{span}\left(\{\tau_k\}_{1 \le k \le p}\right)$; for any $r \le p$, there exists a (not necessarily unique) r-dimensional subspace $\mathcal{S}_r$ of $\mathcal{S}$ such that

$$\sum_{k=1}^{p} \min_{h_k \in \mathcal{S}_r} \|\tau_k - h_k\|^2 \le \sum_{k=1}^{p} \min_{v_k \in \mathcal{V}_r} \|\tau_k - v_k\|^2 , \tag{6}$$

for any r-dimensional subspace $\mathcal{V}_r$ of $\mathcal{S}$. The optimal subspace $\mathcal{S}_r$ can be found by assembling the covariance operator

$$\mathcal{C} = \sum_{k=1}^{p} \tau_k \otimes \tau_k = \sum_{k=1}^{p} \tau_k \langle \tau_k, \cdot \rangle , \tag{7}$$

and taking the sum of the eigenspaces associated with the r largest eigenvalues. Note that because $\mathcal{C}$ is self-adjoint, the eigendecomposition coincides with the singular value decomposition.

Numerically, the computation of these eigenfunctions can be carried out in two ways. The more obvious approach relies on assembling a discretization of the covariance operator and performing its eigendecomposition. More precisely, defining the $M \times p$ matrix A by $A_{ik} = \tau_k(x_i)$ allows us to build $C_M = AA^*W$, an approximation to $\mathcal{C}$. Its eigendecomposition then yields the eigenfunctions evaluated at the quadrature nodes $\{x_i\}_{1 \le i \le M}$. However, this approach is prohibitive in practice because it requires assembling a large square matrix whose dimensions scale with the number of quadrature points M and therefore the dimension d. As a result, the complexity of the eigendecomposition procedure scales cubically with the number of quadrature points.

Defining the operators $\mathcal{A} : \mathbb{C}^p \to \mathcal{S}$ and $\mathcal{A}^* : \mathcal{S} \to \mathbb{C}^p$ by

$$\mathcal{A}\mathbf{b} = \sum_{k=1}^{p} b_k \tau_k, \qquad (\mathcal{A}^* f)_k = \langle \tau_k, f \rangle , \text{ for } 1 \le k \le p, \tag{8}$$

allows us to write $\mathcal{C} = \mathcal{A}\mathcal{A}^*$. Let

$$\mathcal{A} = \sum_{k=1}^{p} \sigma_k \phi_k \mathbf{v}_k^* \tag{9}$$

be the singular value decomposition (SVD) of $\mathcal{A}$; here, $\sigma_1 \ge \sigma_2 \ge \ldots \ge \sigma_p > 0$ are the singular values, $\{\phi_k\}_{1 \le k \le p} \subset \mathcal{S}$ is a collection of functions orthonormal with respect to $\langle \cdot, \cdot \rangle$, and

$\{\mathbf{v}_k\}_{1\le k\le p} \subset \mathbb{C}^p$ is a set of orthonormal vectors with respect to the Euclidean inner product. This leads to

$$\mathcal{C} = \sum_{k=1}^{p} \sigma_k^2 \left(\phi_k \otimes \phi_k\right), \tag{10}$$

thus demonstrating that the desired eigenfunctions of $\mathcal{C}$ are simply the $\{\phi_k\}$.

The alternative approach to finding $\mathcal{S}_r$ relies on the observation that knowledge of the singular values $\{\sigma_k\}$ and right singular vectors $\{\mathbf{v}_k\}$, together with the $\{\tau_k\}$, is sufficient for computing the $\{\phi_k\}$. This can be accomplished by defining the Gram matrix $\mathcal{D} = \mathcal{A}^*\mathcal{A}$; this is a $p \times p$ matrix comprising the pairwise inner products $\mathcal{D}_{kl} = \langle \tau_k, \tau_l\rangle$. Note then that $\mathcal{D} = VS^2V^*$, where V and S are $p \times p$ matrices given by $V_{lk} = (\mathbf{v}_k)_l$ and $S = \operatorname{diag}(\sigma_1, \ldots, \sigma_p)$. The discretization

$$\mathcal{D} \approx D_M := A^*WA \tag{11}$$

allows the calculation of (approximate) V and S via the SVD and thus the recovery of $\{\phi_k\}_{1\le k\le p}$ by

$$\phi_k = \sigma_k^{-1} \sum_{l=1}^{p} (\mathbf{v}_k)_l\, \tau_l. \tag{12}$$

However, this prescription relies on division by singular values that may decay rapidly, so the corresponding orthonormal basis calculations can suffer from large errors. In addition, assembling the matrix $\mathcal{D}$ explicitly squares the singular values, with the result that we can only compute them with accuracy on the order of the square root of machine precision. The latter shortcoming can be overcome by instead making use of the "square root" of $\mathcal{D}$, given by $B_M = W^{1/2}A$, and computing its SVD $B_M = QSV^*$.

Supplementary Figure 4 shows the results of applying this procedure to the trunk net functions obtained from training the periodic advection problem $u_t + u_x = 0$ on $\Omega = [0, 2\pi]$. Evaluating the temporal trunk net functions at $t = 0$ yields $p = 128$ spatial functions, the first few of which are shown in Supplementary Figure 4a. From the graphs in Supplementary Figure 4b, it appears that successive eigenfunctions undulate with increasing frequency, suggesting that this family possesses a natural frequency-based hierarchy. Also note that, barring the first three functions, the zeros of successive eigenfunctions separate each other in the manner of orthogonal polynomials.

In addition, using (7) and (10), we have

$$\sigma_l^2 = \langle \phi_l, \mathcal{C}\phi_l\rangle = \sum_{k=1}^{p} \left|\langle \tau_k, \phi_l\rangle\right|^2, \tag{13}$$

thus establishing that the singular values serve as a gauge of the contribution of each eigenfunction to $\mathcal{S}$. The exponentially decaying singular values in Supplementary Figure 4c are therefore not only indicative of a hierarchy, but also suggest that below a certain threshold, the corresponding eigenfunctions are essentially noise. As a result, the initial few eigenfunctions (until around ϕ_{60} in this example) are, for all practical purposes, sufficient for representing $\mathcal{S}$. However, because the ratio of the largest and smallest singular values gives the condition number, we deduce that B_M (and thus A) has a large condition number. This indicates that any procedure that directly makes use of the $\{\tau_k\}$ (i.e., up to a change of basis) is likely to be ill-conditioned.

One instance of this can be seen in Supplementary Figure 4d, where the magnitudes of the expansion coefficients $a_k = \langle \phi_k, f\rangle$ for $f(x) = e^{\sin(x)}$ are shown. The steep decay is interrupted at $k \approx 40$, and the values start increasing. As mentioned earlier, this is a consequence of using (12):

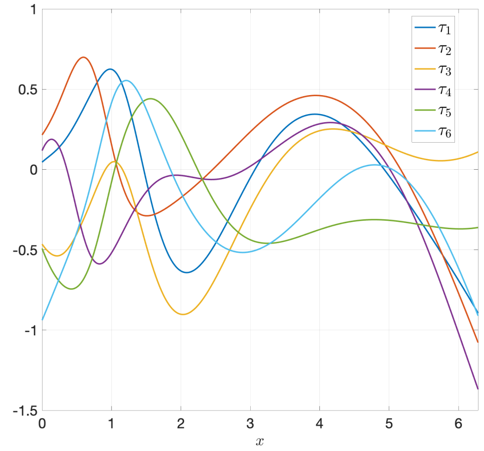

(a) The first few trunk net functions

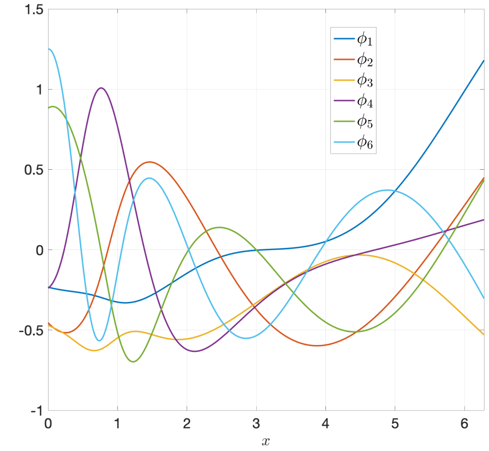

(b) The first few eigenfunctions

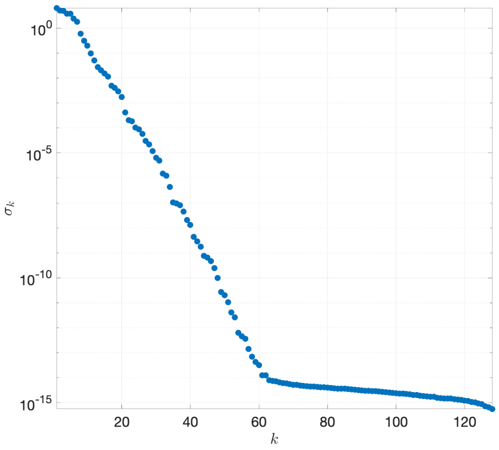

(c) The singular values $\{\sigma_k\}_{1 \leq k \leq p}$

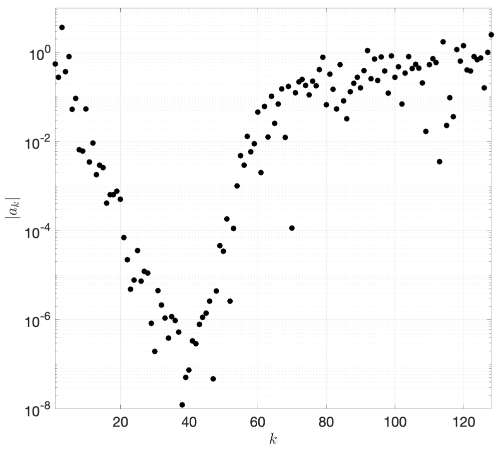

(d) Expansion coefficients for $e^{\sin(x)}$

Supplementary Figure 4: Results for the trunk net functions obtained from training the periodic advection problem $u_t + u_x = 0$ on $\Omega = [0, 2\pi]$ frozen at $t = 0$. We used $M = 2^{10}$ Gauss–Legendre quadrature points and employed the "square root" formulation with (12) to compute the eigenfunctions.

the round-off error in the calculation of ϕ_k, and thus a_k, is roughly $\epsilon\sigma_k^{-1}$, where ϵ is the machine precision, and swamps the expansion coefficient values whenever σ_k goes below a certain value.

These results call for a technique that avoids making use of (12) altogether. One way of achieving this is to utilize the orthogonal matrix Q obtained from the SVD of B_M. Note that the entries of $W^{-1/2}Q$ provide the values of the eigenfunctions at the quadrature points via

$$\phi_k(x_i) = (W^{-1/2}Q)_{ik} \text{ for } 1 \le i \le M \text{ and } 1 \le k \le p. \tag{14}$$

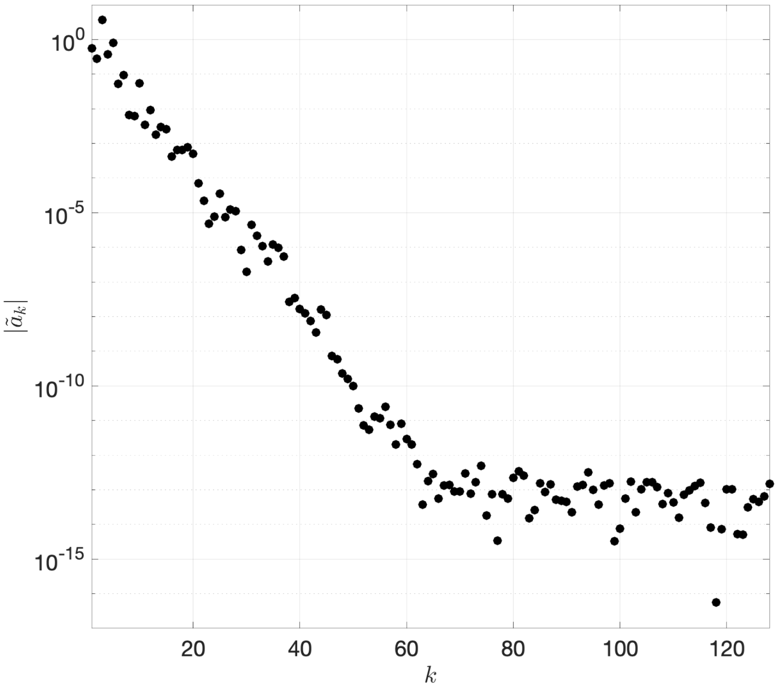

Supplementary Figure 5: The expansion coefficient magnitudes for $f(x) = e^{\sin(x)}$ in terms of the $\{\tilde{\phi}_k\}$ computed using (14) and (15) with $L = 127$.

To allow the evaluation of the $\{\phi_k\}$ at points away from the quadrature grid, we can employ either a local interpolation procedure or an orthogonal polynomial expansion. The choice of Gauss–Legendre quadrature points as the $\{x_i\}$ makes the latter option more appealing. For any $L < M$, let $\{q_j\}_{0\le j\le L}$ be the orthonormal Legendre polynomials on $[0, 2\pi]$ and define the functions $\{\tilde{\phi}_k\}_{1\le k\le p}$ by

$$\tilde{\phi}_k = \sum_{j=0}^{L} \left(\sum_{i=1}^{M} q_j(x_i)\phi_k(x_i)\omega_i \right) q_j. \tag{15}$$

Observe that for $L = M-1$, this is equivalent to a polynomial interpolation through $\{\phi_k(x_i)\}_{1\le i\le M}$. Because the $\{\phi_k\}$ are smooth, this polynomial expansion is guaranteed to be highly accurate. By choosing L large enough, we can be fairly confident that the $\{\tilde{\phi}_k\}$ serve as good approximations to $\{\phi_k\}$. More significantly, the procedure only makes use of operations (14) and (15), both of which are well-conditioned. This is demonstrated in Supplementary Figure 5, where the magnitudes of the expansion coefficients $\tilde{a}_k = \left\langle \tilde{\phi}_k, f \right\rangle$ for $f(x) = e^{\sin(x)}$ can be seen to decay all the way to machine

precision (using $L = 127$). This shows that SVD orthonormalization followed by a polynomial expansion forms an accurate and stable method to obtain a hierarchical basis from the trunk net functions.

Most of the ingredients used above easily generalize to higher dimensions and complex domains. However, one obvious pitfall is that higher-dimensional analogs of Legendre expansions are not readily available on arbitrary domains. This poses a challenge to our ability to evaluate the orthonormal eigenfunctions away from the quadrature grid points if we forgo the use of (12). However, several approaches can be used to bridge this gap, chief among which is a local spline-based interpolation method. Similarly, one can also utilize the recent partition of unity networks proposed in [5, 6] that solve the regression problem on complex geometries by computing a partition of unity coupled with high-order polynomial expansions supported on the different partitions. Yet another approach is to use an extension algorithm to extend the eigenfunctions to larger, more regular domains where Legendre expansions are available. That such extensions exist in principle follows from the Whitney extension theorem [7, 8], while several methods have been proposed to carry out this procedure in practice, e.g., [9, 10, 11, 12]. These methodologies will be further explored, and the results presented in a future publication.

3 Assessing the approximation capability of the custom-made basis functions

The sharp decay in the expansion coefficients of the initial conditions (e.g., in Supplementary Figure 5) suggests that the orthonormal basis functions $\{\phi_k\}_{1\leq k\leq p}$ are highly adept at approximating smooth functions. In this section, we investigate this capability more fully. In contrast with the DeepONet analyses [13, 14], we study the properties of a trained trunk net function space and, in doing so, account for estimation and optimization errors in addition to the approximation errors.

For any function $f : [0, 2\pi] \to \mathbb{C}$, we can define the orthogonal projection

$$\mathcal{P}f := \sum_{k=1}^{p} \langle \phi_k, f\rangle \, \phi_k. \tag{16}$$

It follows that

$$\|f - \mathcal{P}f\| = \min_{g\in\mathcal{S}} \|f - g\| \,. \tag{17}$$

Let $\{q_j\}_{j\geq 0}$ be the orthonormal Legendre polynomials on $[0, 2\pi]$ and, for any $r \geq 0$, let

$$\mathcal{L}_r f = \sum_{j=0}^{r} \langle q_j, f\rangle \, q_j \tag{18}$$

denote the Legendre expansion of f up to q_r. Because $\mathcal{P}\mathcal{L}_r f \in \mathcal{S}$, we have from (17)

$$\|f - \mathcal{P}f\| \leq \|f - \mathcal{P}\mathcal{L}_r f\| \,, \tag{19}$$

and thus

$$\|f - \mathcal{P}f\| \leq \|f - \mathcal{L}_r f\| + \|\mathcal{L}_r f - \mathcal{P}\mathcal{L}_r f\| \,. \tag{20}$$

From Parseval's theorem, we have

$$\|f - \mathcal{L}_r f\|^2 = \sum_{j\geq r+1} |\langle q_j, f\rangle|^2, \tag{21}$$

while (18) yields

$$\|\mathcal{L}_r f - \mathcal{P}\mathcal{L}_r f\| = \left\| \sum_{j=0}^{r} \langle q_j, f\rangle \,(q_j - \mathcal{P}q_j) \right\| \leq \sum_{j=0}^{r} |\langle q_j, f\rangle| \, \|q_j - \mathcal{P}q_j\| \,. \tag{22}$$

Plugging (21) and (22) into (20) yields

$$\|f - \mathcal{P}f\| \leq \left(\sum_{j\geq r+1} |\langle q_j, f\rangle|^2 \right)^{1/2} + \sum_{j=0}^{r} |\langle q_j, f\rangle| \, \|q_j - \mathcal{P}q_j\| \,. \tag{23}$$

Thus, we can bound the approximation error of an arbitrary function in terms of the Legendre expansion coefficients and the errors made in approximating Legendre polynomials using our custom basis. Recall that for smooth f, the Legendre expansion coefficients $\langle q_j, f\rangle$ decay rapidly. Therefore, for sufficiently large r, we only need to focus on the second term in (23).

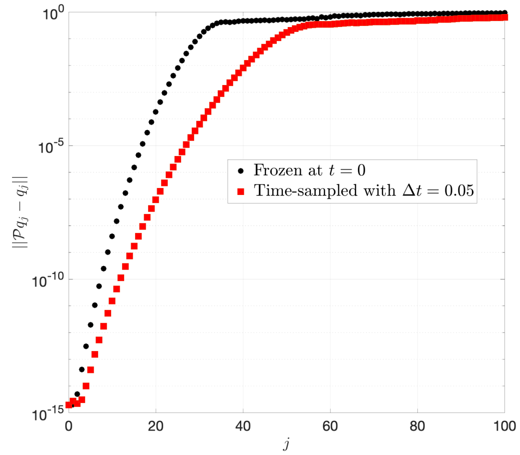

(a)

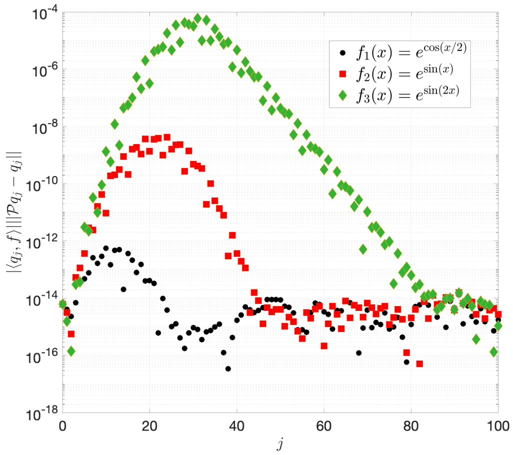

(b)

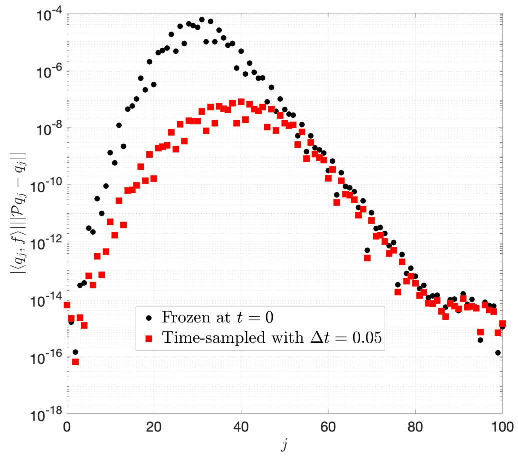

(c)

Supplementary Figure 6: (a) Errors in the approximation of the jth Legendre polynomial using the custom basis functions derived from the DeepONet trained for the periodic advection problem $u_t + u_x = 0$ on $[0, 2\pi]$. The errors appear to increase exponentially, indicating the difficulty in approximating high-frequency functions. The growth is markedly slower when the basis functions are drawn from the larger collection of time-sampled trunk net functions. (b) Terms in the upper bound (23) computed for functions with varying frequencies using the $t = 0$ trunk net functions. Low-frequency functions possess rapidly decaying Legendre coefficients and are therefore able to damp the growth seen in (a); this becomes harder with increasing frequency. (c) The upper bound terms in (23) for $f(x) = e^{\sin(2x)}$, shown for both cases considered in (a). The slower growth associated with the time-sampled trunk functions leads to much smaller error bounds, helping explain the lower errors in Supplementary Table 4.

Supplementary Figure 6a shows that the errors $\|q_j - \mathcal{P}q_j\|$ in approximating Legendre polynomials using the basis functions considered in Section 2 (derived from the DeepONet trained on the advection equation $u_t + u_x = 0$) increase exponentially with j before leveling off. Along with the basis functions derived from the trunk net frozen at $t = 0$, we present the results from sampling the trunk net at equispaced values in the temporal domain (we refer to these as the time-sampled functions). Using a trunk net of width $w = 128$ as before and choosing the time-step size $\Delta t = 0.05$, we obtain $p = (1 + 1/\Delta t)w = 2688$ trunk net functions from which the basis functions are drawn. For this case, we use $M = 2^{12}$ Gauss–Legendre quadrature nodes; to allow for a fair comparison with the $t = 0$ case, we employ 128 basis functions for both tests. The growth is markedly slower in the time-sampled case, suggesting that it is better equipped to approximate high-frequency functions. The plateauing of the errors in both cases is an expected outcome of using an architecture based on the UAT for operators because it is only valid for compact subsets of $C(\Omega)$ and therefore cannot account for arbitrarily large frequencies. Nevertheless, this diagram goes some way toward assessing the completeness properties of the custom basis functions by showing the errors in approximating successive elements of a complete polynomial basis.

Because these errors appear in tandem with the decaying Legendre expansion coefficients in (23), we can expect this steep growth to be damped. This is illustrated in Supplementary Figure 6b, where the $|\langle q_j, f\rangle|\,\|q_j - \mathcal{P}q_j\|$ have been computed for $f_1(x) = e^{\cos(x/2)}$, $f_2(x) = e^{\sin(x)}$, and $f_3(x) = e^{\sin(2x)}$. It can be seen that the low-frequency f_1 possesses rapidly decaying Legendre coefficients and are therefore more capable of damping the growth seen in Supplementary Figure 6a; for increasing frequencies, this becomes a much more difficult task. Finally, in Supplementary Figure 6c, we compare the upper bound terms from the $t = 0$ trunk net functions and the time-sampled ones for f_3. Due to the superior capability of the latter at approximating higher-order Legendre polynomials, the upper bound terms are much smaller, indicating that the actual errors $\|f - \mathcal{P}f\|$ would also be lower. That this is indeed the case generally is confirmed in Supplementary Table 4.

$f(x)$	Frozen at $t = 0$	Time-sampled
$e^{\cos(x/2)}$	9.14×10^{-15}	1.38×10^{-14}
$e^{\sin(x)}$	9.91×10^{-13}	1.29×10^{-14}
$e^{\sin(2x)}$	3.67×10^{-8}	4.66×10^{-14}

Supplementary Table 4: The errors $\|f - \mathcal{P}f\|$ computed for functions with increasing frequencies on $[0, 2\pi]$, using the custom basis functions derived from trunk net functions frozen at $t = 0$ as well as from those sampled at time values spaced apart at $\Delta t = 0.05$. To account for the much larger dimension of the space in the latter case, we only make use of the first 128 basis functions. The time-sampled approach generally yields much smaller errors, indicating the scope of this approach.

4 Applying boundary conditions for non-periodic basis functions

In this section, we detail our procedure for imposing boundary conditions in a Galerkin method when the basis functions are non-periodic. Our approach relies on ideas developed in the context of discontinuous Galerkin (DG) methods. In the following sections, the appropriate boundary condition treatment is presented for the one-dimensional advection (4.1), diffusion (4.2), inviscid Burgers (4.3), reduced Korteweg–de Vries (4.4), and reduced Kuramoto–Sivashinsky (4.5) equations on periodic domains. The problems considered in the main paper can then be accommodated by combining these basic treatments.

4.1 Advection equation

Consider the one-dimensional advection equation with periodic boundary conditions

$$\frac{\partial u}{\partial t} + \alpha \frac{\partial u}{\partial x} = 0, \quad t > 0, \; x \in [0, 2\pi], \tag{24}$$

where the choice of α determines the flow direction. We begin by applying the Galerkin condition

$$\left\langle \phi_m, \frac{\partial u}{\partial t} + \alpha \frac{\partial u}{\partial x} \right\rangle = 0, \quad \text{for } 1 \leq m \leq r, \tag{25}$$

integrating by parts, and rewriting the resulting flux term as a surface integral that yields

$$\left\langle \phi_m, \frac{\partial u}{\partial t} \right\rangle = \left\langle \alpha \frac{\partial}{\partial x} \phi_m, u \right\rangle - \int_{\partial\Omega} (\hat{\boldsymbol{n}} \cdot \alpha)\, \phi_m(s) u_B(s) \; ds, \tag{26}$$

where $\hat{\boldsymbol{n}}$ is the outward facing normal and u_B is the appropriate choice of u at the boundary. Let $x_{\text{in}} = 0$ and $x_{\text{out}} = 2\pi$ for $\alpha \geq 0$ and $x_{\text{in}} = 2\pi$ and $x_{\text{out}} = 0$ for $\alpha < 0$. Simplifying the surface integral in (26), expanding $u^r(t,x) = \sum_{k=1}^{r} a_k(t)\phi_k(x)$, and setting $u_B = u(x_{\text{out}})$ to incorporate upwinding then yields a system of ordinary differential equations (ODEs) for the expansion coefficients [15]

$$\frac{da_m(t)}{dt} = \sum_{k=1}^{r} \left[\alpha \left\langle \frac{d}{dx} \phi_m, \phi_k \right\rangle - |\alpha| \left(\phi_m(x_{\text{out}}) - \phi_m(x_{\text{in}}) \right) \phi_k(x_{\text{out}}) \right] a_k(t). \tag{27}$$

The initial condition for this system of equations is inferred from the initial condition $u(0,x)$ by

$$a_m(0) = \langle \phi_m, u(0, \cdot) \rangle, \quad 1 \leq m \leq r. \tag{28}$$

In the case of a Dirichlet boundary condition $u(x_{\text{in}}) = u_{\text{in}}$, we simply plug it in at the corresponding boundary section in (26) to obtain

$$\frac{da_m(t)}{dt} = \sum_{k=1}^{r} \left[\alpha \left\langle \frac{d}{dx} \phi_m, \phi_k \right\rangle - |\alpha| \phi_m(x_{\text{out}}) \phi_k(x_{\text{out}}) \right] a_k(t) + |\alpha| \phi_m(x_{\text{in}}) u_{\text{in}}. \tag{29}$$

4.2 Diffusion equation

Consider the one-dimensional diffusion equation with periodic boundary conditions given by

$$\frac{\partial u}{\partial t} - \nu \frac{\partial^2 u}{\partial x^2} = 0, \quad t > 0, \; x \in [0, 2\pi], \tag{30}$$

where ν is the viscosity. We begin by rewriting (30) as a system of first-order equations

$$\frac{\partial u}{\partial t} - \nu \frac{\partial q}{\partial x} = 0, \quad q - \frac{\partial u}{\partial x} = 0, \tag{31}$$

and applying the Galerkin condition to the two equations to get

$$\left\langle \phi_k, \frac{\partial u}{\partial t} - \nu \frac{\partial q}{\partial x} \right\rangle = 0, \quad \left\langle \phi_k, q - \frac{\partial u}{\partial x} \right\rangle = 0. \tag{32}$$

Integrating each equation by parts and rewriting the resulting flux term as a surface integral yields

$$\begin{aligned} \left\langle \phi_k, \frac{\partial u}{\partial t} \right\rangle &= -\nu \left\langle \frac{\partial}{\partial x}\phi_k, q \right\rangle + \nu \int_{\partial\Omega} \hat{\boldsymbol{n}} \cdot q_B(s)\phi_k(s)\, ds, \\ \langle \phi_k, q \rangle &= -\left\langle \frac{\partial}{\partial x}\phi_k, u \right\rangle + \int_{\partial\Omega} \hat{\boldsymbol{n}} \cdot u_B(s)\phi_k(s) ds, \end{aligned} \tag{33}$$

where $\hat{\boldsymbol{n}}$ is the outward-facing normal, q_B is the appropriate choice of q at the boundary, and u_B is the appropriate choice of u at the boundary. Expanding the surface integrals, utilizing the expansions in terms of the custom basis functions $u^r(t,x) = \sum_{i=1}^r a_i(t)\phi_i(x)$, $q^r(t,x) = \sum_{j=1}^r b_j(t)\phi_j(x)$, and incorporating the periodic boundary conditions [16, 15] yields

$$\frac{da_m(t)}{dt} = \nu \sum_{j=1}^r \left[-\left\langle \frac{d}{dx}\phi_m, \phi_j \right\rangle + (\phi_m(x_{\text{out}}) - \phi_m(x_{\text{in}}))\phi_j(x_{\text{out}}) \right] b_j(t), \tag{34}$$

$$b_m(t) = \sum_{i=1}^r \left[-\left\langle \frac{d}{dx}\phi_m, \phi_i \right\rangle + (\phi_m(x_{\text{out}}) - \phi_m(x_{\text{in}}))\phi_i(x_{\text{in}}) \right] a_i(t). \tag{35}$$

Combining Equations (34) and (35) yields a system of ODEs for the expansion coefficients

$$\begin{aligned} \frac{da_m(t)}{dt} = \nu \sum_{j=1}^r &\left[-\left\langle \frac{d}{dx}\phi_m, \phi_j \right\rangle + (\phi_m(x_{\text{out}}) - \phi_m(x_{\text{in}}))\phi_j(x_{\text{out}}) \right] \cdot \\ &\sum_{i=1}^r \left[-\left\langle \frac{d}{dx}\phi_j, \phi_i \right\rangle + (\phi_j(x_{\text{out}}) - \phi_j(x_{\text{in}}))\phi_i(x_{\text{in}}) \right] a_i(t), \end{aligned} \tag{36}$$

where $x_{\text{in}} = 0$ and $x_{\text{out}} = 2\pi$, and the initial condition for this system of equations is inferred from the initial condition $u(0,x)$ by (28).

The decomposition (31) also enables the imposition of Dirichlet boundary conditions. Assuming (30) is complemented by $u(t,0) = u_L(t)$ and $u(t,2\pi) = u_R(t)$, we obtain, instead of (33),

$$\begin{aligned} \left\langle \phi_k, \frac{\partial u}{\partial t} \right\rangle &= -\nu \left\langle \frac{\partial}{\partial x}\phi_k, q \right\rangle + \nu \int_{\partial\Omega} \hat{\boldsymbol{n}} \cdot q(s)\phi_k(s)\, ds, \\ \langle \phi_k, q \rangle &= -\left\langle \frac{\partial}{\partial x}\phi_k, u \right\rangle + \int_{\partial\Omega} \hat{\boldsymbol{n}} \cdot u_B(s)\phi_k(s) ds. \end{aligned} \tag{37}$$

Plugging in the expansions, replacing u_B with the provided boundary conditions, and combining the two systems as above yields the ODE system

$$\begin{aligned} \frac{da_m(t)}{dt} = \nu \sum_{j=1}^r &\left[-\left\langle \frac{d}{dx}\phi_m, \phi_j \right\rangle + \phi_m(x_{\text{out}})\phi_j(x_{\text{out}}) - \phi_m(x_{\text{in}})\phi_j(x_{\text{in}}) \right] \cdot \\ &\left[-\sum_{i=1}^r \left\langle \frac{d}{dx}\phi_j, \phi_i \right\rangle a_i(t) + \phi_j(x_{\text{out}})u_R(t) - \phi_j(x_{\text{in}})u_L(t) \right]. \end{aligned} \tag{38}$$

4.3 Inviscid Burgers equation

Consider the inviscid Burgers equation with periodic boundary conditions

$$\frac{\partial u}{\partial t} + \frac{\partial}{\partial x}\left(\frac{u^2}{2}\right) = 0, \quad x \in [0, 2\pi]. \tag{39}$$

We set $f(u) = u^2/2$ and begin by applying the Galerkin condition

$$\left\langle \phi_k, \frac{\partial u}{\partial t} + \frac{\partial u}{\partial x}\left(f(u)\right) \right\rangle = 0, \tag{40}$$

integrating by parts, and rewriting the resulting flux term as a surface integral that yields

$$\left\langle \phi_k, \frac{\partial u}{\partial t} \right\rangle = \left\langle \frac{\partial}{\partial x}\phi_k, f(u) \right\rangle - \int_{\partial\Omega} \hat{\boldsymbol{n}} \cdot f^* \phi_k(s)\, ds, \tag{41}$$

where $\hat{\boldsymbol{n}}$ is the outward-facing normal and f^* represents the numerical flux term. Expanding the surface integral in (41) yields a system of ODEs for the expansion coefficients

$$\frac{da_m(t)}{dt} = \left\langle \frac{\partial}{\partial x}\phi_m, f(u) \right\rangle - f^*(u(t, 2\pi), u(t, 0))(\phi_m(2\pi) - \phi_m(0)), \tag{42}$$

where the initial condition for this system of equations is inferred from the initial condition $u(0, x)$ by (28). The numerical flux $f^*(u^-, u^+)$ must obey the following properties to convey the correct information across the interface [17]:

- consistency: $f^*(u, u) = f(u)$
- continuity: f^* must be at least Lipschitz continuous with respect to both arguments
- monotonicity: f^* must be non-decreasing in the first argument and non-increasing in the second.

Among others, examples of such fluxes include the Lax–Friedrichs flux

$$f^*_{\mathrm{LF}}(u^-, u^+) = \frac{1}{2}\left(f(u^-) + f(u^+) - \alpha(u^+ - u^-)\right), \quad \alpha = \max_u |f'(u)|, \tag{43}$$

where the maximum is taken over the values of u on Ω, and the Godunov flux

$$f^*_{\mathrm{G}}(u^-, u^+) = \begin{cases} \min_{u^- \le u \le u^+} f(u), & \text{if } u^- < u^+ \\ \max_{u^+ \le u \le u^-} f(u), & \text{if } u^+ \le u^-. \end{cases} \tag{44}$$

We, however, employ

$$f^*_A(u^-, u^+) = f\left(\frac{u^- + |u^-|}{2} - \frac{u^+ - |u^+|}{2}\right), \tag{45}$$

which possesses the property

$$f^*_A(u^-, u^+) = \begin{cases} f(u^-), & \text{if } u^+ \ge 0, \\ f(u^+), & \text{if } u^- \le 0. \end{cases} \tag{46}$$

This choice has the benefit of not requiring a maximum or minimum value of f over an interval, unlike (43) and (44)), and therefore leads to faster computations. In addition, it clearly obeys the consistency and continuity properties; for monotonicity, we note that

- if $u^+ \geq 0$, then from (46) f_A^* is increasing in u^- if $u^- > 0$ and non-decreasing if $u^- \leq 0$;
- if $u^- \leq 0$, then from (46) f_A^* is decreasing in u^+ if $u^+ < 0$ and non-increasing if $u^+ \geq 0$;
- if $u^+ < 0$ and $u^- > 0$, then

$$\frac{u^- + |u^-|}{2} - \frac{u^+ - |u^+|}{2} = u^- - u^+ \Rightarrow f_A^*(u^-, u^+) = \frac{1}{2}(u^- - u^+)^2 \tag{47}$$

from (45) so that

$$\frac{\partial f_A^*}{\partial u^-}(u^-, u^+) = u^- - u^+ > 0, \qquad \frac{\partial f_A^*}{\partial u^+}(u^-, u^+) = -(u^- - u^+) < 0. \tag{48}$$

4.4 Reduced Korteweg–de Vries equation

Consider the reduced Korteweg–de Vries equation with periodic boundary conditions

$$\frac{\partial u}{\partial t} + \delta^2 \frac{\partial^3 u}{\partial x^3} = 0, \quad x \in [0, 2\pi], \tag{49}$$

where δ is the strength of dispersion. We begin by rewriting (49) as a system of first-order equations

$$\frac{\partial u}{\partial t} + \delta^2 \frac{\partial q}{\partial x} = 0, \;\; q - \frac{\partial p}{\partial x} = 0, \;\; p - \frac{\partial u}{\partial x} = 0, \tag{50}$$

and applying the Galerkin condition to the three equations to obtain

$$\left\langle \phi_k, \frac{\partial u}{\partial t} + \delta^2 \frac{\partial q}{\partial x} \right\rangle = 0, \quad \left\langle \phi_k, q - \frac{\partial p}{\partial x} \right\rangle = 0, \quad \left\langle \phi_k, p - \frac{\partial u}{\partial x} \right\rangle = 0. \tag{51}$$

Integrating each equation by parts and rewriting the resulting flux term as a surface integral yields

$$\begin{aligned} \left\langle \phi_k, \frac{\partial u}{\partial t} \right\rangle &= \delta^2 \left\langle \frac{\partial}{\partial x}\phi_k, q \right\rangle - \delta^2 \int_{\partial\Omega} \hat{\boldsymbol{n}} \cdot q_B(s)\phi_k(s)\; ds, \\ \langle \phi_k, q \rangle &= - \left\langle \frac{\partial}{\partial x}\phi_k, p \right\rangle + \int_{\partial\Omega} \hat{\boldsymbol{n}} \cdot p_B(s)\phi_k(s) ds, \\ \langle \phi_k, p \rangle &= - \left\langle \frac{\partial}{\partial x}\phi_k, u \right\rangle + \int_{\partial\Omega} \hat{\boldsymbol{n}} \cdot u_B(s)\phi_k(s) ds, \end{aligned} \tag{52}$$

where $\hat{\boldsymbol{n}}$ is the outward-facing normal, q_B is the appropriate choice of q at the boundary, p_B is the appropriate choice of p at the boundary, and u_B is the appropriate choice of u at the boundary. Expanding the surface integrals, utilizing the expansions in terms of the custom basis functions $u^r(t,x) = \sum_{i=1}^r a_i(t)\phi_i(x)$, $q^r(t,x) = \sum_{j=1}^r b_j(t)\phi_j(x)$, $p^r(t,x) = \sum_{l=1}^r c_l(t)\phi_l(x)$, and incorporating the periodic boundary conditions [18] yields

$$\frac{da_m(t)}{dt} = \delta^2 \sum_{j=1}^r \left[\left\langle \frac{d}{dx}\phi_m, \phi_j \right\rangle - (\phi_m(x_{\text{out}}) - \phi_m(x_{\text{in}}))\phi_j(x_{\text{in}}) \right] b_j(t), \tag{53}$$

$$b_m(t) = \sum_{l=1}^r \left[- \left\langle \frac{d}{dx}\phi_m, \phi_l \right\rangle + (\phi_m(x_{\text{out}}) - \phi_m(x_{\text{in}}))\phi_l(x_{\text{in}}) \right] c_l(t), \tag{54}$$

$$c_m(t) = \sum_{i=1}^r \left[- \left\langle \frac{d}{dx}\phi_m, \phi_i \right\rangle + (\phi_m(x_{\text{out}}) - \phi_m(x_{\text{in}}))\phi_i(x_{\text{out}}) \right] a_i(t). \tag{55}$$

Combining equations (53), (54), and (55) yields a system of ODEs for the expansion coefficients,

$$\frac{da_m(t)}{dt} = \delta^2 \sum_{j=1}^{r} \left[\left\langle \frac{d}{dx}\phi_m, \phi_j \right\rangle - (\phi_m(x_{\text{out}}) - \phi_m(x_{\text{in}}))\phi_j(x_{\text{in}}) \right] \cdot \sum_{l=1}^{r} \left[-\left\langle \frac{d}{dx}\phi_j, \phi_l \right\rangle + (\phi_j(x_{\text{out}}) - \phi_j(x_{\text{in}}))\phi_l(x_{\text{in}}) \right] \cdot \sum_{i=1}^{r} \left[-\left\langle \frac{d}{dx}\phi_l, \phi_i \right\rangle + (\phi_l(x_{\text{out}}) - \phi_l(x_{\text{in}}))\phi_i(x_{\text{out}}) \right] a_i(t), \tag{56}$$

with $x_{\text{in}} = 0$ and $x_{\text{out}} = 2\pi$, and the initial condition for this system of equations is inferred from the initial condition $u(0, x)$ by (28).

4.5 Reduced Kuramoto–Sivashinsky equation

Consider the reduced Kuramoto–Sivashinsky equation with periodic boundary conditions

$$\frac{\partial u}{\partial t} + \beta \frac{\partial^4 u}{\partial x^4} = 0, \tag{57}$$

where β is the viscosity. We begin by rewriting (57) as a system of first-order equations

$$\frac{\partial u}{\partial t} + \beta \frac{\partial q}{\partial x} = 0, \;\; q - \frac{\partial p}{\partial x} = 0, \;\; p - \frac{\partial w}{\partial x} = 0, \;\; w - \frac{\partial u}{\partial x} = 0, \tag{58}$$

and applying the Galerkin condition to the four equations

$$\left\langle \phi_k, \frac{\partial u}{\partial t} + \beta \frac{\partial q}{\partial x} \right\rangle = 0, \;\; \left\langle \phi_k, q - \frac{\partial p}{\partial x} \right\rangle = 0, \;\; \left\langle \phi_k, p - \frac{\partial w}{\partial x} \right\rangle = 0, \;\; \left\langle \phi_k, w - \frac{\partial u}{\partial x} \right\rangle = 0. \tag{59}$$

Integrating each equation by parts and rewriting the resulting flux term as a surface integral yields

$$\begin{aligned} \left\langle \phi_k, \frac{\partial u}{\partial t} \right\rangle &= \beta \left\langle \frac{\partial}{\partial x}\phi_k, q \right\rangle - \beta \int_{\partial\Omega} \hat{\boldsymbol{n}} \cdot q_B(s)\phi_k(s)\; ds, \\ \langle \phi_k, q \rangle &= -\left\langle \frac{\partial}{\partial x}\phi_k, p \right\rangle + \int_{\partial\Omega} \hat{\boldsymbol{n}} \cdot p_B(s)\phi_k(s) ds, \\ \langle \phi_k, p \rangle &= -\left\langle \frac{\partial}{\partial x}\phi_k, w \right\rangle + \int_{\partial\Omega} \hat{\boldsymbol{n}} \cdot w_B(s)\phi_k(s) ds, \\ \langle \phi_k, w \rangle &= -\left\langle \frac{\partial}{\partial x}\phi_k, u \right\rangle + \int_{\partial\Omega} \hat{\boldsymbol{n}} \cdot u_B(s)\phi_k(s) ds, \end{aligned} \tag{60}$$

where $\hat{\boldsymbol{n}}$ is the outward-facing normal, q_B is the appropriate choice of q at the boundary, p_B is the appropriate choice of p at the boundary, w_B is the appropriate choice of w at the boundary, and u_B is the appropriate choice of u at the boundary. Expanding the surface integrals, utilizing the expansions in terms of the custom basis functions $u^r(t,x) = \sum_{i=1}^{r} a_i(t)\phi_i(x)$, $q^r(t,x) = \sum_{j=1}^{r} b_j(t)\phi_j(x)$, $p^r(t,x) = \sum_{l=1}^{r} c_l(t)\phi_l(x)$, $w^r(t,x) = \sum_{z=1}^{r} d_z(t)\phi_z(x)$, and incorporating the periodic boundary

conditions [19] leads to

$$\frac{da_m(t)}{dt} = \beta \sum_{j=1}^{r} \left[\left\langle \frac{d}{dx}\phi_m, \phi_j \right\rangle - (\phi_m(x_{\text{out}}) - \phi_m(x_{\text{in}}))\phi_j(x_{\text{in}}) \right] b_j(t), \tag{61}$$

$$b_m(t) = \sum_{l=1}^{r} \left[-\left\langle \frac{d}{dx}\phi_m, \phi_l \right\rangle + (\phi_m(x_{\text{out}}) - \phi_m(x_{\text{in}}))\phi_l(x_{\text{out}}) \right] c_l(t), \tag{62}$$

$$c_m(t) = \sum_{z=1}^{r} \left[-\left\langle \frac{d}{dx}\phi_m, \phi_z \right\rangle + (\phi_m(x_{\text{out}}) - \phi_m(x_{\text{in}}))\phi_z(x_{\text{out}}) \right] d_z(t), \tag{63}$$

$$d_m(t) = \sum_{i=1}^{r} \left[-\left\langle \frac{d}{dx}\phi_m, \phi_i \right\rangle + (\phi_m(x_{\text{out}}) - \phi_m(x_{\text{in}}))\phi_i(x_{\text{in}}) \right] a_i(t). \tag{64}$$

Combining equations (61), (62), (63), and (64) yields a system of ODEs for the expansion coefficients

$$\begin{aligned} \frac{da_m(t)}{dt} = \beta \sum_{j=1}^{r} &\left[\left\langle \frac{d}{dx}\phi_m, \phi_j \right\rangle - (\phi_m(x_{\text{out}}) - \phi_m(x_{\text{in}}))\phi_j(x_{\text{in}}) \right] \cdot \\ \sum_{l=1}^{r} &\left[-\left\langle \frac{d}{dx}\phi_j, \phi_l \right\rangle + (\phi_j(x_{\text{out}}) - \phi_j(x_{\text{in}}))\phi_l(x_{\text{out}}) \right] \cdot \\ \sum_{z=1}^{r} &\left[-\left\langle \frac{d}{dx}\phi_l, \phi_z \right\rangle + (\phi_l(x_{\text{out}}) - \phi_l(x_{\text{in}}))\phi_z(x_{\text{out}}) \right] \cdot \\ \sum_{i=1}^{r} &\left[-\left\langle \frac{d}{dx}\phi_z, \phi_i \right\rangle + (\phi_z(x_{\text{out}}) - \phi_z(x_{\text{in}}))\phi_i(x_{\text{in}}) \right] a_i(t), \end{aligned} \tag{65}$$

with $x_{\text{in}} = 0$ and $x_{\text{out}} = 2\pi$, and the initial condition for this system of equations is inferred from the initial condition $u(0, x)$ by (28).

5 Implementation details for time integration of ODE systems resulting from using custom-made basis functions

The custom bases were constructed as in Section 2 of this Supplement with $L = 127$ (see (15)) for all examples except the inviscid Burgers equation, which utilized $L = 1629$. The trunk net functions were sampled at $t = 0$ for all PDEs. Recall that if the singular value corresponding to a basis function is too small, the function itself is predominantly noise. As a result, the number r of basis functions used to solve the PDE is specified to be the functions with a corresponding singular value larger than 10^{-13}. This can be seen as a form of model reduction without the memory term.

All the examples, with the exception of the inviscid Burgers equation (2448-node), are solved on a 128-node Gauss–Legendre quadrature grid to facilitate the usage of this highly accurate scheme for calculating inner products. The differentiation of basis functions was performed using automatic differentiation for all examples except the inviscid Burgers equation, where differentiation of basis functions was performed using the Legendre projection and the derivatives of the Legendre polynomials. The quadratic nonlinear terms are computed in modal space, while all the necessary triple product integrals were computed beforehand. The systems of ODEs for the expansion coefficients of the advection, advection-diffusion, viscous Burgers, and inviscid Burgers equations with periodic boundary conditions were integrated in time using the adaptive step size, Runge–Kutta–Dormand–Prince integrator. The solution was saved at time values of 10^{-3} apart for the linear and 10^{-4} apart for the nonlinear PDEs. The solution for the inviscid Burgers equation and the temporal domain $t \in [0, 250]$ was saved at values of 10^{-2} apart. The relative and absolute error tolerances were specified as 10^{-10} and 10^{-14}, respectively. The system of ODEs for the expansion coefficients of the advection-diffusion equation with Dirichlet boundary conditions was integrated in time using an explicit, singly diagonal, implicit Runge–Kutta integrator with adaptive step size, relative error tolerance 10^{-10}, absolute error tolerance 10^{-14}, and with the solution saved at time values of 10^{-3} apart. The system of ODEs for the expansion coefficients of the Korteweg–de Vries equation was integrated in time using an explicit, singly diagonal, implicit Runge–Kutta integrator with adaptive step size. The system of ODEs for the expansion coefficients of the Kuramoto–Sivashinsky equation was integrated in time using a Crank–Nicolson integrator with adaptive step size. The relative and absolute error tolerances for the Korteweg–de Vries and Kuramoto–Sivashinsky equations were specified as 10^{-8} and 10^{-12}, respectively. The solutions were saved at time values of 10^{-4} apart.

The error presented for each PDE is a relative Euclidean two-norm error defined by

$$E(t) = \frac{\|u^r(t,\cdot) - u_{\mathrm{G}}(t,\cdot)\|}{\|u_{\mathrm{G}}(t,\cdot)\|}, \tag{66}$$

where u_{G} is the ground truth solution computed using a Fourier expansion, Legendre polynomial expansion, or MUSCL scheme, and u^r is the custom basis function solution using r basis functions. When comparing against a Fourier solution, the $M = 128$ or $M = 512$ Fourier expansion coefficients and (2) are used to approximate the solution at the non-uniform quadrature nodes. In the case of MUSCL, the solution computed on the spatial grid consisting of 4096 discretization points is interpolated using piecewise linear interpolation to approximate the solution at each of the non-uniform quadrature nodes. The error presented for the inviscid Burgers equation and the temporal domain $t \in [0, 250]$ was computed using solution data saved at values of 10^{-2} apart.

6 Additional results

In this section, we display results that have been omitted from the main text due to space constraints. First, we consider the advection equation

$$u_t + \alpha u_x = 0, \quad t > 0, \; x \in [0, 2\pi], \tag{67}$$

with periodic boundary conditions and initial condition $u(0, x) = u_0(x)$. Using 53 custom basis functions with $\alpha = 1$, we obtain the spatiotemporal plots shown in Supplementary Figures 7, 8, and 9. The corresponding error plots are shown in Supplementary Figure 10.

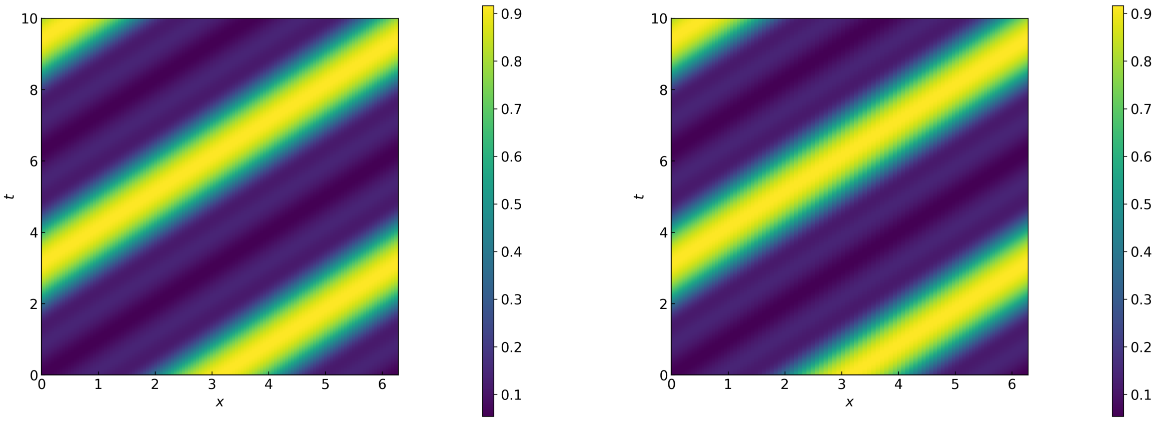

(a) The Fourier solution (b) Custom basis solution

Supplementary Figure 7: Advection spatiotemporal plots for the random in-distribution initial condition.

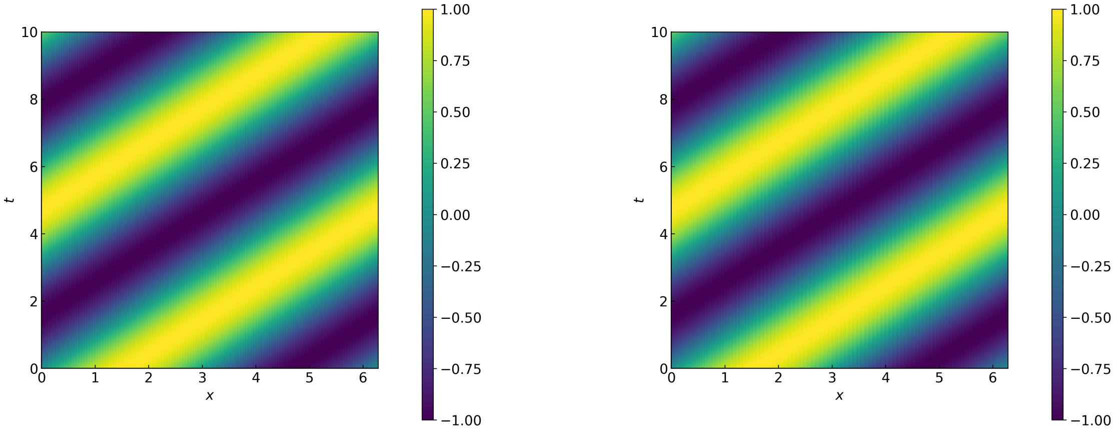

(a) The Fourier solution (b) Custom basis solution

Supplementary Figure 8: Advection spatiotemporal plots for $u_0(x) = \sin(x)$.

Next, we turn to the advection-diffusion problem. The spatiotemporal plots for the initial conditions considered in the main text are displayed in Supplementary Figures 11, 12, and 13.

To further illustrate the flexibility of our method, we can use it to solve problems with parameters different from those used for training. In Supplementary Figures 14, 15, 16, and 17, we show

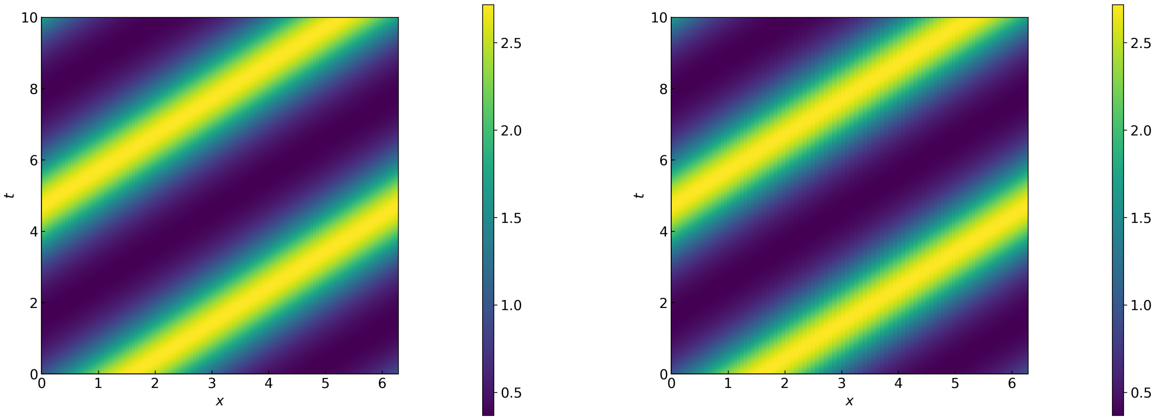

(a) The Fourier solution

(b) Custom basis solution

Supplementary Figure 9: Advection spatiotemporal plots for $u_0(x) = e^{\sin(x)}$.

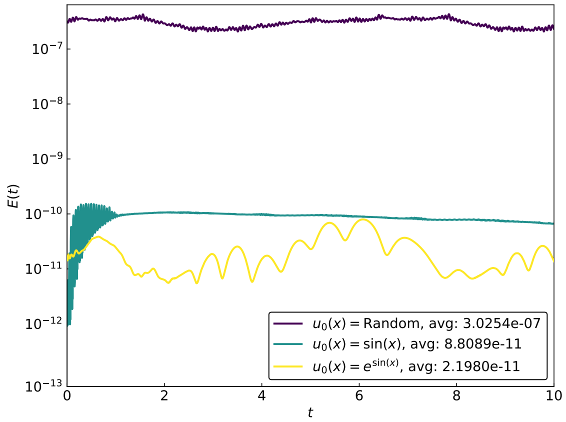

Supplementary Figure 10: Relative errors from using the custom basis functions to solve the advection equation.

the spatiotemporal plots and evolution errors for the advection-diffusion equation with $\alpha = 4$ and $\nu = 0.01$.

Continuing with the advection-diffusion equation, but with Dirichlet rather than periodic boundary conditions. The spatiotemporal plots for the initial conditions considered in the main text when using custom basis functions identified for the period problem are displayed in Supplementary Figures 18, 19, and 20, while the spatiotemporal plots when using custom basis functions explicitly identified for the Dirichlet problem are displayed in Supplementary Figures 21, 22, and 23.

Moving on to the nonlinear problems, the spatiotemporal plots for the out-of-distribution initial conditions $u_0(x) = \sin(x)$ and $u_0(x) = e^{\sin(x)}$ are presented in Supplementary Figures 24, 25, and 26, and the energy decay plots for the inviscid Burgers equation for $u_0(x) = \sin(x)$ and the in-distribution initial condition are shown in Supplementary Figure 27.

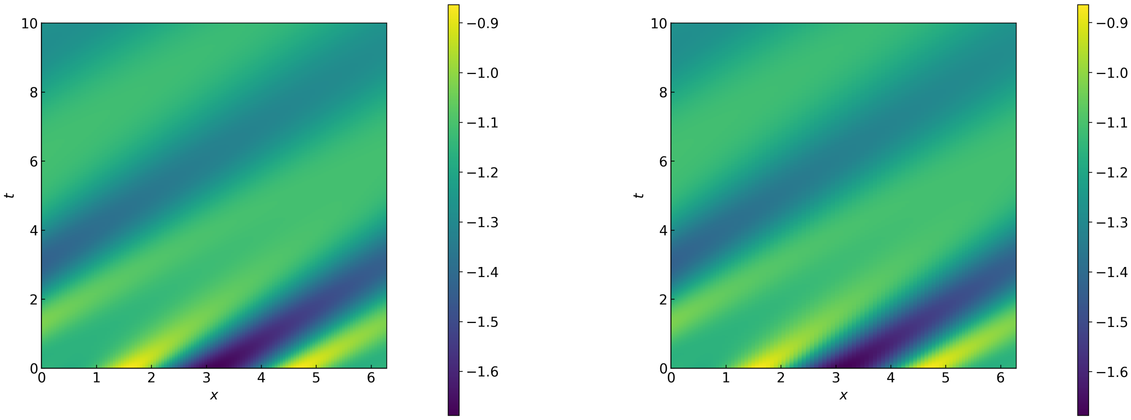

(a) The Fourier solution (b) Custom basis solution

Supplementary Figure 11: Advection-diffusion spatiotemporal plots for the random in-distribution initial condition.

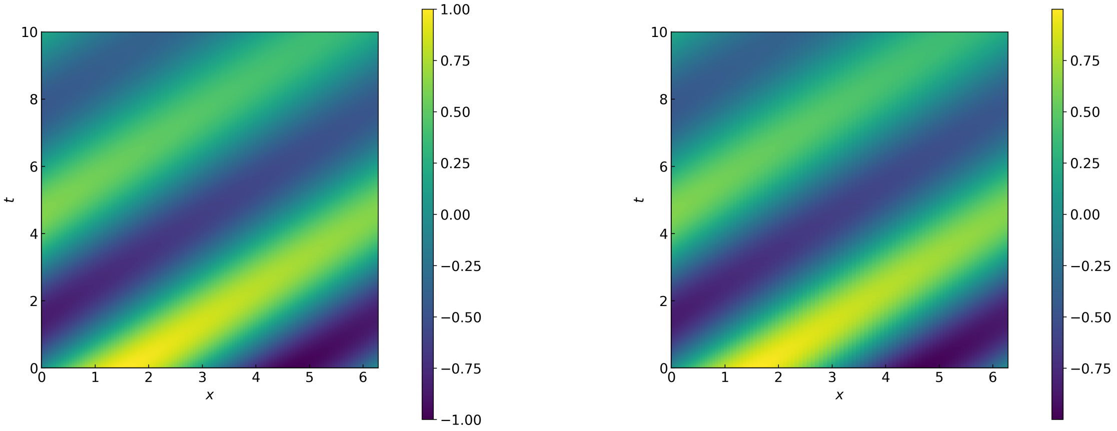

(a) The Fourier solution (b) Custom basis solution

Supplementary Figure 12: Advection-diffusion spatiotemporal plots for $u_0(x) = \sin(x)$.

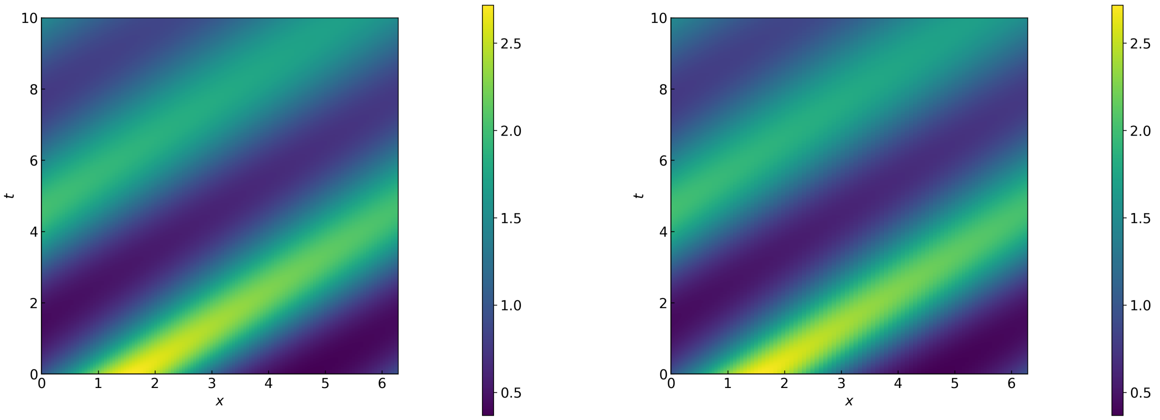

(a) The Fourier solution

(b) Custom basis solution

Supplementary Figure 13: Advection-diffusion spatiotemporal plots for $u_0(x) = e^{\sin(x)}$.

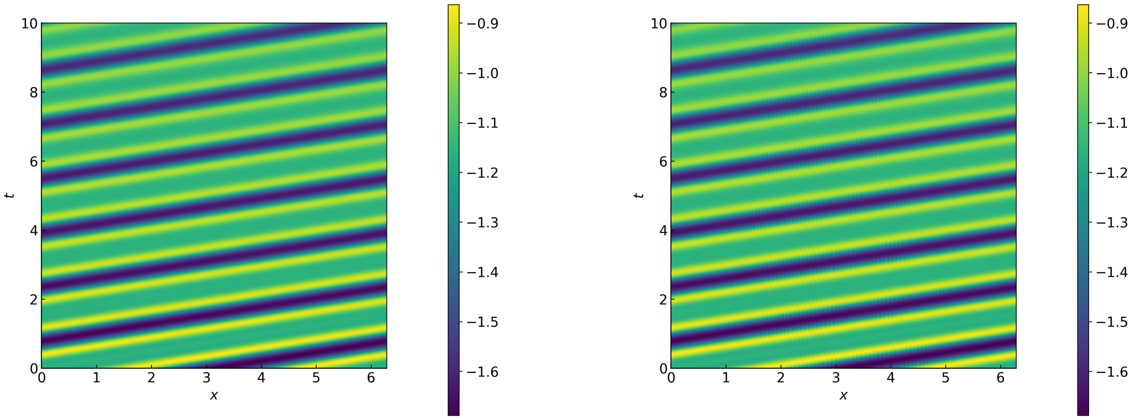

(a) The Fourier solution

(b) Custom basis solution

Supplementary Figure 14: Advection-diffusion spatiotemporal plots for the random in-distribution initial condition with $\alpha = 4$ and $\nu = 0.01$.

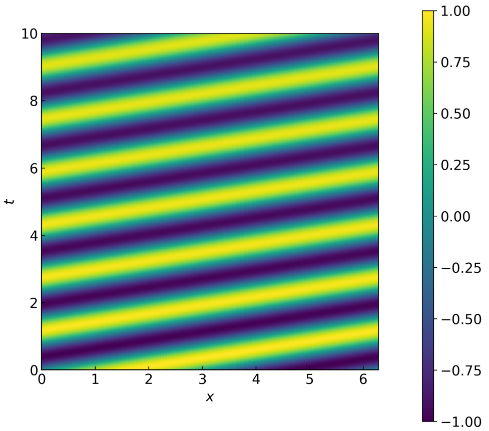

(a) The Fourier solution

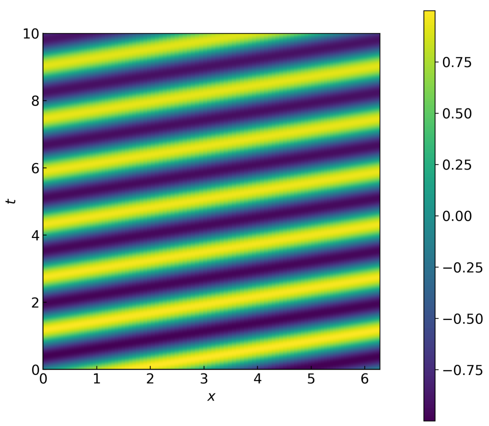

(b) Custom basis solution

Supplementary Figure 15: Advection-diffusion spatiotemporal plots for $u_0(x) = \sin(x)$ with $\alpha = 4$ and $\nu = 0.01$.

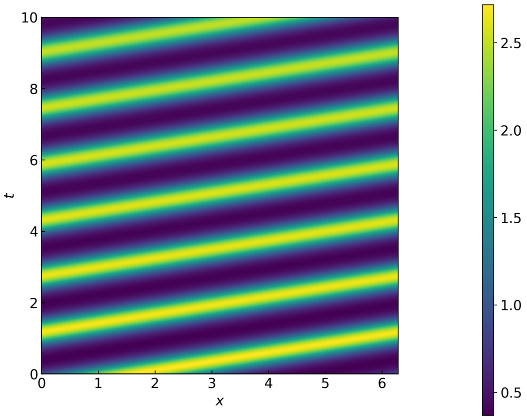

(a) The Fourier solution

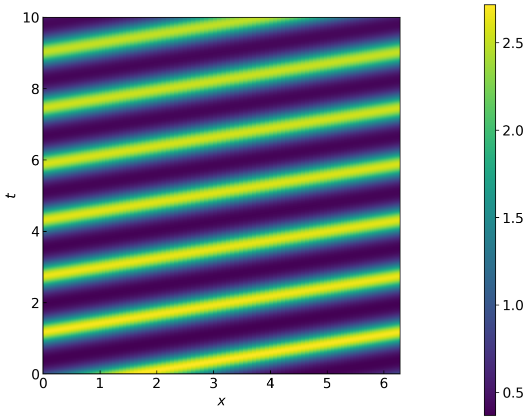

(b) Custom basis solution

Supplementary Figure 16: Advection-diffusion spatiotemporal plots for $u_0(x) = e^{\sin(x)}$ with $\alpha = 4$ and $\nu = 0.01$.

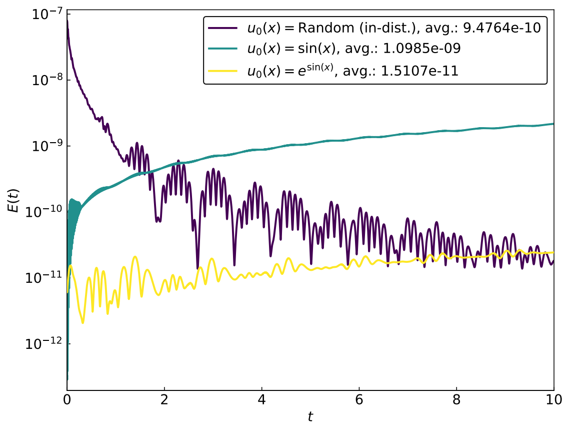

Supplementary Figure 17: Relative errors from using the custom basis functions to solve the advection-diffusion equation with $\alpha = 4$ and $\nu = 0.01$.

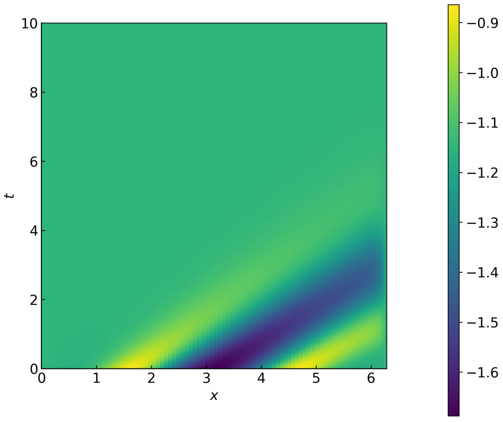

(a) The Legendre solution

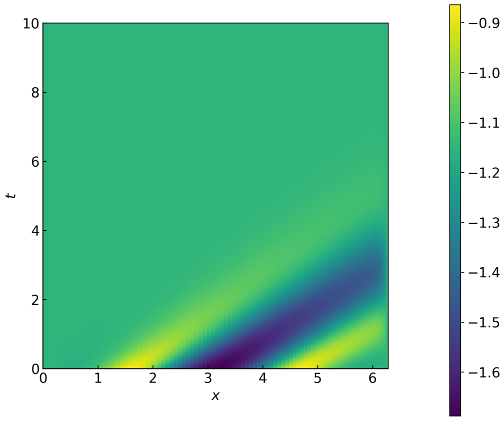

(b) Custom basis solution

Supplementary Figure 18: Advection-diffusion spatiotemporal plots for the random in-distribution initial condition and Dirichlet boundary conditions when using custom basis functions identified for the periodic problem.

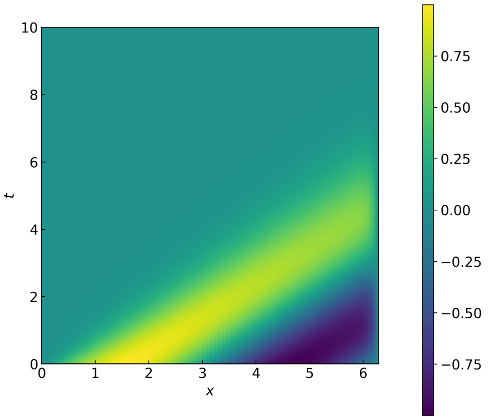

(a) The Legendre solution

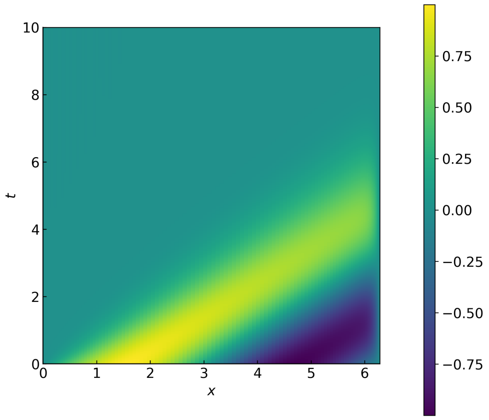

(b) Custom basis solution

Supplementary Figure 19: Advection-diffusion spatiotemporal plots for $u_0(x) = \sin(x)$ and Dirichlet boundary conditions when using custom basis functions identified for the periodic problem.

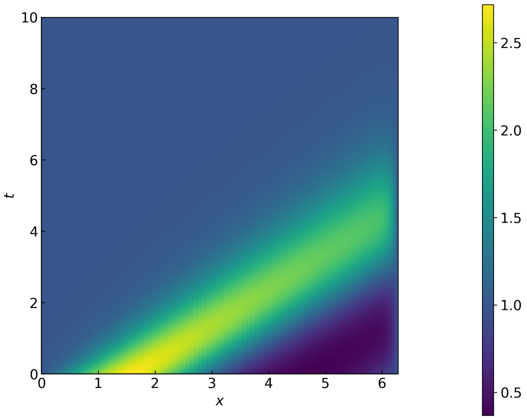

(a) The Legendre solution

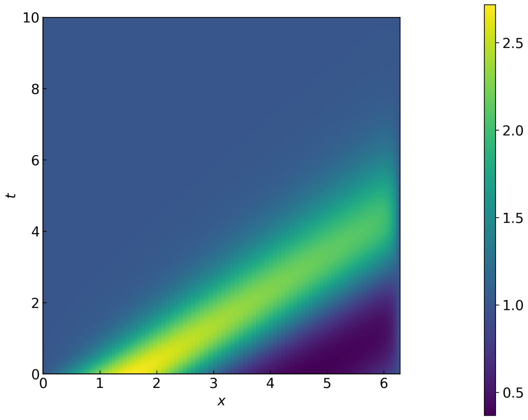

(b) Custom basis solution

Supplementary Figure 20: Advection-diffusion spatiotemporal plots for $u_0(x) = e^{\sin(x)}$ and Dirichlet boundary conditions when using custom basis functions identified for the periodic problem.

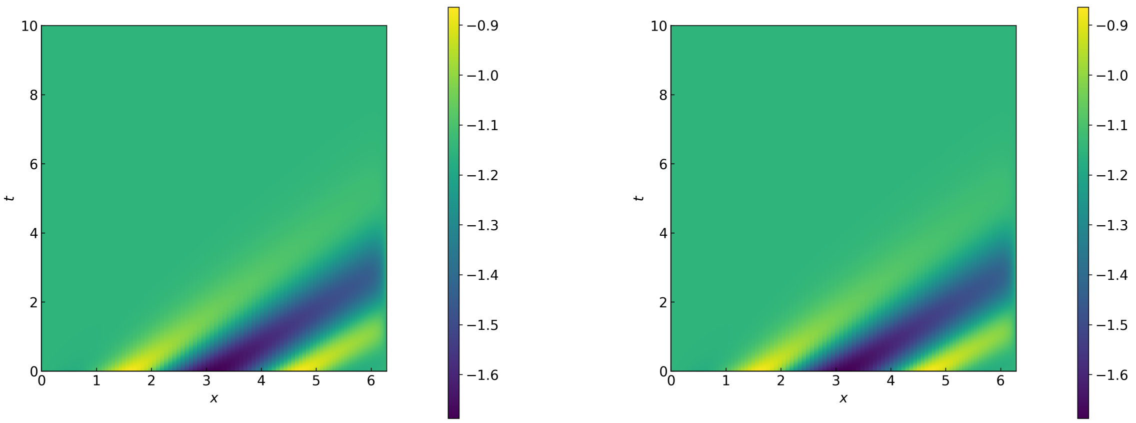

(a) The Legendre solution

(b) Custom basis solution

Supplementary Figure 21: Advection-diffusion spatiotemporal plots for the random in-distribution initial condition and Dirichlet boundary conditions when using custom basis functions explicitly identified for the Dirichlet problem.

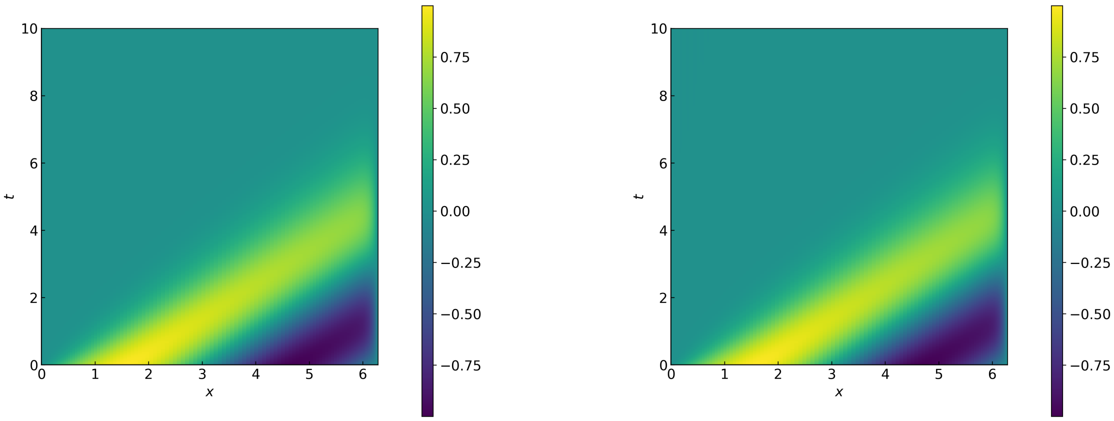

(a) The Legendre solution

(b) Custom basis solution

Supplementary Figure 22: Advection-diffusion spatiotemporal plots for $u_0(x) = \sin(x)$ and Dirichlet boundary conditions when using custom basis functions explicitly identified for the Dirichlet problem.

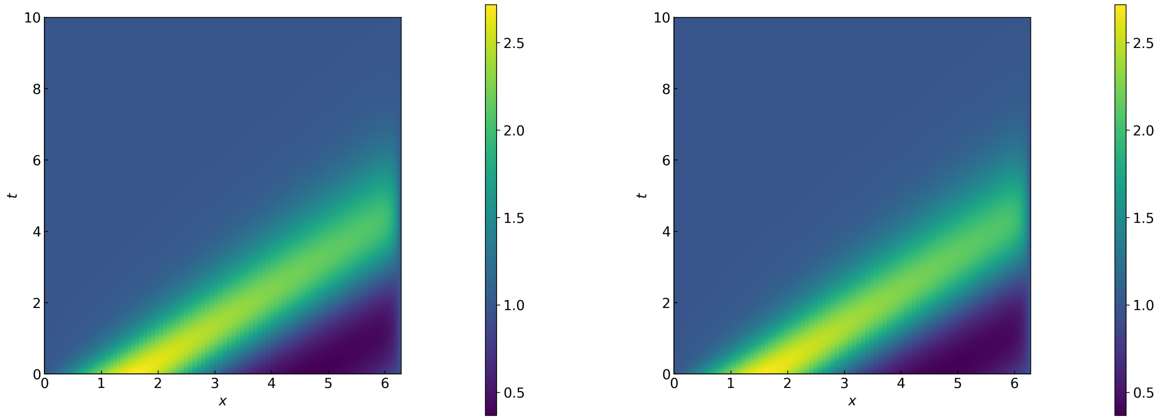

(a) The Legendre solution

(b) Custom basis solution

Supplementary Figure 23: Advection-diffusion spatiotemporal plots for $u_0(x) = e^{\sin(x)}$ and Dirichlet boundary conditions when using custom basis functions explicitly identified for the Dirichlet problem.

Mean wall-clock times for each of the six models are shown in Supplementary Tables 5, 6, 7, 8, 9, 10, and 11. The time presented for the advection, advection-diffusion, viscous Burgers, Korteweg–de Vries, and Kuramoto–Sivashinsky equations is the time required to numerically integrate the r, L, or M size system of ordinary differential equations corresponding to the expansion or modal coefficients for 10 units of time. For the Fourier solution, conjugacy was not exploited and all modal coefficients (positive and negative) were evolved. The mean wall-clock time presented for the inviscid Burgers equation is the time required to numerically integrate the r size system of ordinary differential equations corresponding to the expansion coefficients or the MUSCL solution for the stated spatial discretization for 10 units of time. The in-distribution initial condition was used to initialize the models, the mean was calculated based on three runs (post initial compilation run), and all tests were conducted on an Intel Core i9-9900K CPU.

The errors presented for the Fourier and Legendre solutions are based on the same 128-node Gauss–Legendre quadrature grid used for the custom basis function solutions. For the nonlinear models, custom basis function results are shown for two different treatments of the nonlinear term; the triple product integral which computes the nonlinear term in the expansion coefficient space, and a pseudo-spectral transform which transforms the expansion coefficient solution to real space, performs the required multiplication, and then returns the solution to the expansion coefficient space. The transformation from expansion coefficient to real space is achieved by directly calculating the series expansion, while the transformation from real to expansion coefficient space is achieved by directly calculating the necessary inner products. This pseudo-spectral transform represents a preliminary step toward reducing the wall-clock times for nonlinear problems solved using custom basis functions.

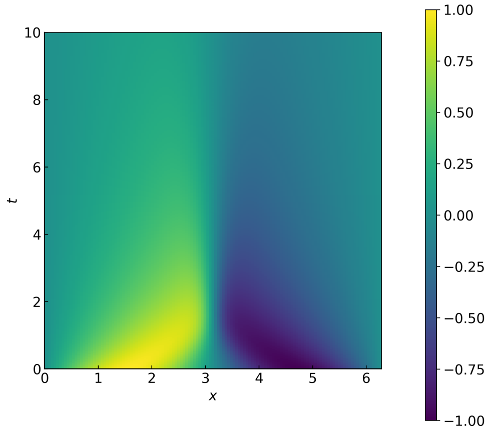

(a) The Fourier solution

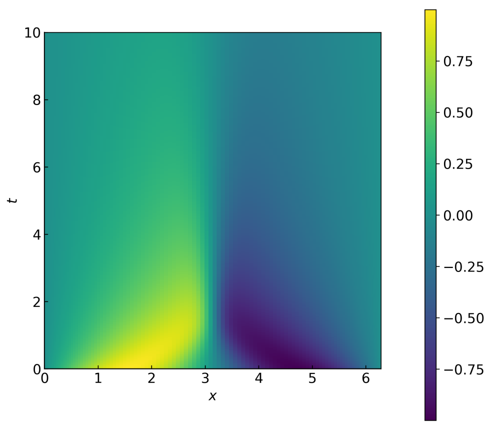

(b) Custom basis solution

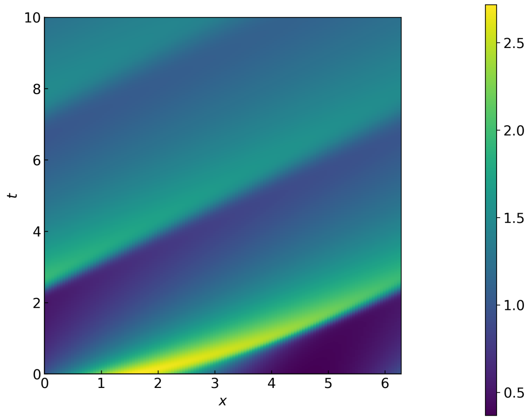

(c) The Fourier solution

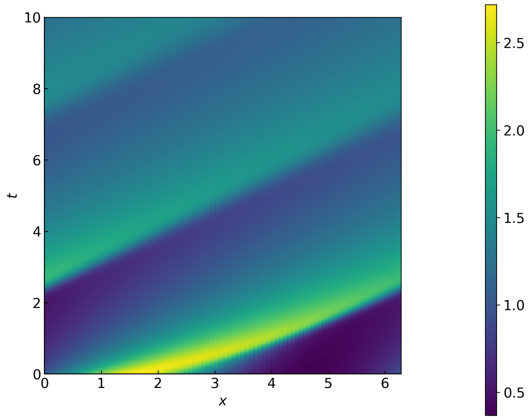

(d) Custom basis solution

Supplementary Figure 24: Results for the viscous Burgers equation for (a,b) $u_0(x) = \sin(x)$ and (c,d) $u_0(x) = e^{\sin(x)}$.

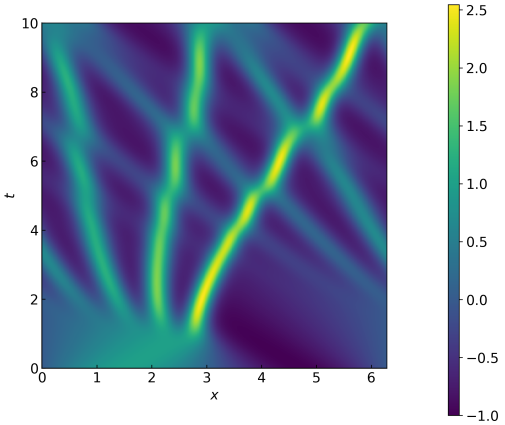

(a) The Fourier solution

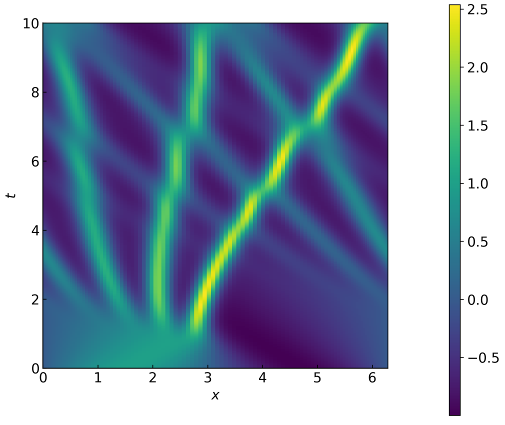

(b) Custom basis solution

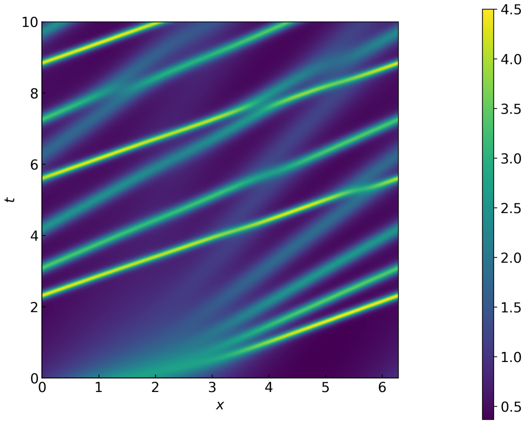

(c) The Fourier solution

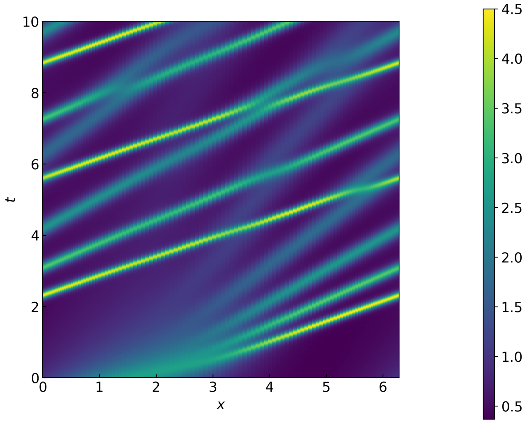

(d) Custom basis solution

Supplementary Figure 25: Results for the Korteweg–de Vries equation for (a,b) $u_0(x) = \sin(x)$ and (c,d) $u_0(x) = e^{\sin(x)}$.

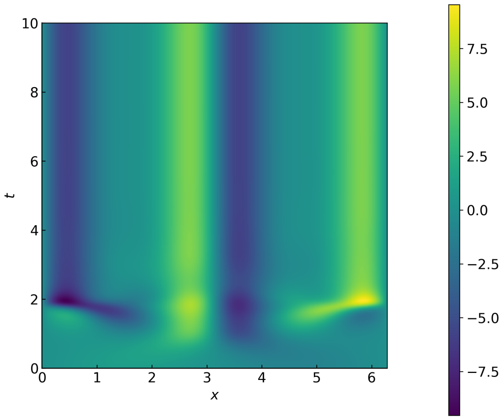

(a) The Fourier solution

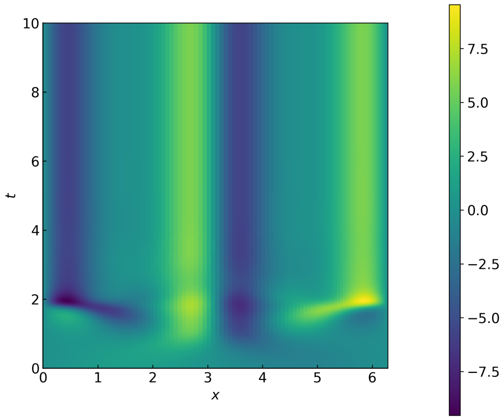

(b) Custom basis solution

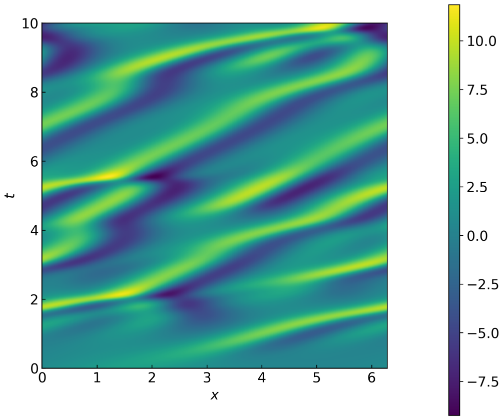

(c) The Fourier solution

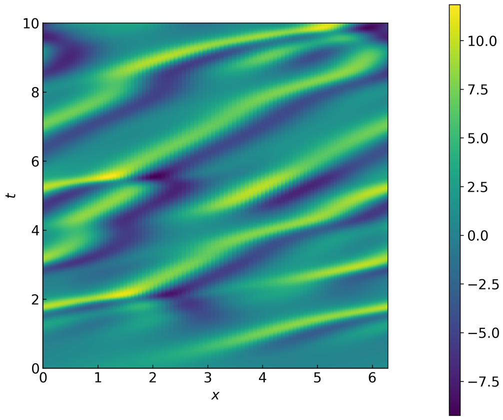

(d) Custom basis solution

Supplementary Figure 26: Results for the Kuramoto–Sivashinsky equation for (a,b) $u_0(x) = \sin(x)$ and (c,d) $u_0(x) = e^{\sin(x)}$.

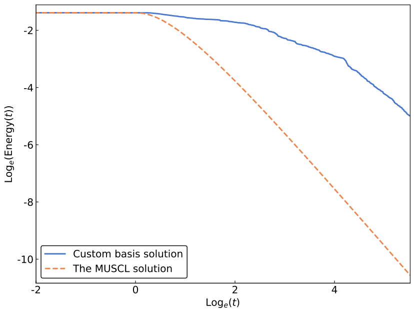

(a) Evolution of the energy

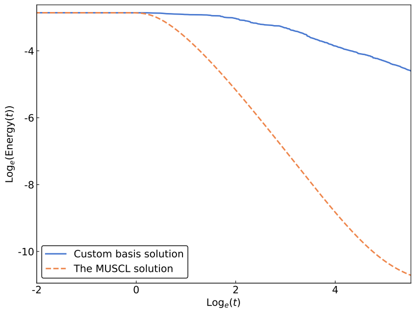

(b) Evolution of the energy

Supplementary Figure 27: Results for the inviscid Burgers equation for (a) $u_0(x) = \sin(x)$ and (b) random in-distribution initial condition.

Solution method	Mean wall-clock time (s)	Average relative error
Custom basis function solution ($r = 53$)	0.007680	3.0254×10^{-7}
Fourier solution ($M = 54$)	0.021379	3.6084×10^{-7}
Fourier reference solution ($M = 128$)	0.040358	–

Supplementary Table 5: Mean wall-clock times in seconds and average relative errors for the advection equation computed using the custom basis function and Fourier solution methods. The average errors were computed for $t \in [0, 10]$.

Solution method	Mean wall-clock time (s)	Average relative error
Custom basis function solution ($r = 59$)	0.293744	7.7971×10^{-11}
Fourier solution ($M = 60$)	0.035615	2.6889×10^{-8}
Fourier reference solution ($M = 128$)	0.120678	–

Supplementary Table 6: Mean wall-clock times in seconds and average relative errors for the advection-diffusion equation computed using the custom basis function and Fourier solution methods. The average errors were computed for $t \in [0, 10]$.

Solution method	Mean wall-clock time (s)	Average relative error
Custom basis function solution ($r = 73$)	0.380920	8.5540×10^{-10}
Legendre solution ($L = 72$)	0.602296	1.0585×10^{-10}
Legendre reference solution ($L = 127$)	1.551408	–

Supplementary Table 7: Mean wall-clock times in seconds and average relative errors for the advection-diffusion equation with Dirichlet boundary conditions computed using the custom basis function and Legendre solution methods. The average errors were computed for $t \in [0, 10]$.

Solution method	Mean wall-clock time (s)	Average relative error
Custom basis function solution ($r = 91$)<br>Calculated using triple product integral	230.190098	1.4225×10^{-8}
Custom basis function solution ($r = 91$)<br>Calculated using pseudo-spectral transform	3.792377	1.4225×10^{-8}
Fourier solution ($M = 92$)	4.035121	9.7757×10^{-11}
Fourier reference solution ($M = 128$)	5.343368	–

Supplementary Table 8: Mean wall-clock times in seconds and average relative errors for the viscous Burgers equation computed using the custom basis function and Fourier solution methods. The average errors were computed for $t \in [0, 10]$.

Solution method	Mean wall-clock time (s)	Average relative error
Custom basis function solution ($r = 106$)<br>Calculated using triple product integral	108.112182	1.8670×10^{-5}
Custom basis function solution ($r = 106$)<br>Calculated using pseudo-spectral transform	1.773402	1.8670×10^{-5}
Fourier solution ($M = 106$)	39.179805	8.0910×10^{-7}
Fourier reference solution ($M = 512$)	235.160790	–

Supplementary Table 9: Mean wall-clock times in seconds and average relative errors for the Korteweg–de Vries equation computed using the custom basis function and Fourier solution methods. The average errors were computed for $t \in [0, 10]$.

Solution method	Mean wall-clock time (s)	Average relative error
Custom basis function solution ($r = 105$)<br>Calculated using triple product integral	3640.652389	7.6522×10^{-4}
Custom basis function solution ($r = 105$)<br>Calculated using pseudo-spectral transform	104.315838	7.6634×10^{-4}
Fourier solution ($M = 106$)	83.722244	3.3127×10^{-4}
Fourier reference solution ($M = 512$)	391.701459	–

Supplementary Table 10: Mean wall-clock times in seconds and average relative errors for the Kuramoto–Sivashinsky equation computed using the custom basis function and Fourier solution methods. The average errors were computed for $t \in [0, 10]$.

Solution method	Mean wall-clock time (s)	Average relative error
Custom basis function solution ($r = 128$)<br>Calculated using triple product integral	162.938098	1.7019×10^{0}
Custom basis function solution ($r = 128$)<br>Calculated using pseudo-spectral transform	6.297149	1.7044×10^{0}
MUSCL solution<br>Discretized using spatial grid of 128 points	0.372881	1.1630×10^{-1}
MUSCL reference solution<br>Discretized using a spatial grid of 4096 points	13.987050	–

Supplementary Table 11: Mean wall-clock times in seconds and average relative errors for the inviscid Burgers equation computed using the custom basis function and MUSCL solution methods. The average errors were computed for $t \in [0, 10]$.

7 Performance of time-sampled basis functions

As in Section 3, the approximation capability of the custom basis is significantly improved by sampling the trunk net functions at various points in the temporal domain. Supplementary Figure 28 shows representative results if this is applied to the advection equation using, as before, a sampling gap $\Delta t = 0.05$ for $t \in [0, 1]$. Keeping all other evolution parameters constant, the algorithm finds 98 custom basis functions with a corresponding singular value greater than 10^{-13}. The usage of time-sampling and the increased set of basis functions (98 vs. 53) for solving the PDE results in a decrease in the average error over the testing interval for the in-distribution initial condition and the initial condition $u(0, x) = e^{\sin(x)}$, while for the out-of-distribution initial condition $u(0, x) = \sin(x)$, we find a slight increase in the average error over the temporal domain.

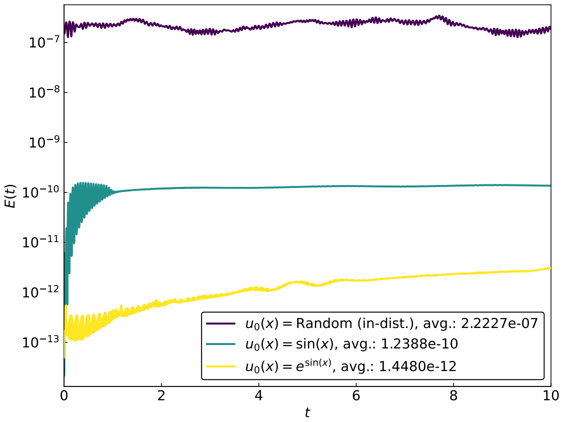

Supplementary Figure 28: Relative errors for the advection equation for $t \in [0, 10]$ and using a time-sampled set of candidate basis functions ($r = 98$).

Supplementary Figure 29 shows the comparison when using the same number of custom basis functions ($r = 53$) taken from $t = 0$ and the time-sampled set of functions. In this case, we find that the latter approach results in a similar average error for the in-distribution initial condition, while for the out-of-distribution problems, we find an increase in the average error over the temporal domain.

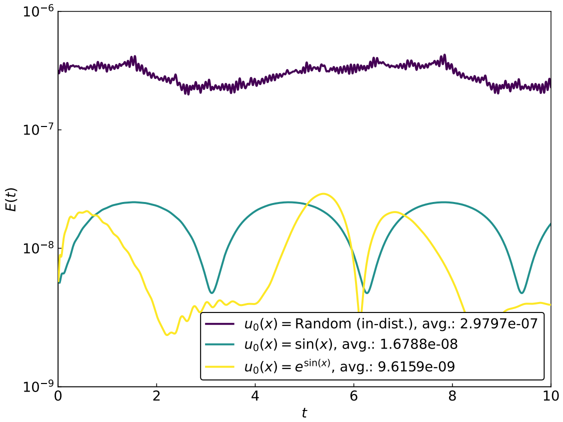

Supplementary Figure 29: Relative errors for the advection equation for $t \in [0, 10]$ and using a time-sampled set of candidate basis functions ($r = 53$).

$u_0(x)$	Frozen at $t = 0$	Time-sampled
Random	3.0254×10^{-7}	2.2227×10^{-7}
$\sin(x)$	8.8089×10^{-11}	1.2388×10^{-10}
$e^{\sin(x)}$	2.1980×10^{-11}	1.4480×10^{-12}

Supplementary Table 12: Average relative errors for the advection equation using the frozen at $t = 0$ ($r = 53$) and the time-sampled ($r = 98$) set of custom basis functions. Average error is computed for $t \in [0, 10]$.

$u_0(x)$	Frozen at $t = 0$	Time-sampled
Random	7.7971×10^{-11}	7.0628×10^{-11}
$\sin(x)$	7.8798×10^{-13}	2.2073×10^{-13}
$e^{\sin(x)}$	8.6461×10^{-13}	1.4576×10^{-13}

Supplementary Table 13: Average relative errors for the advection-diffusion equation using the frozen at $t = 0$ ($r = 59$) and the time-sampled ($r = 87$) set of custom basis functions. Average error is computed for $t \in [0, 10]$.

$u_0(x)$	Frozen at $t = 0$	Time-sampled
Random	1.4225×10^{-8}	1.8992×10^{-6}
$\sin(x)$	9.7880×10^{-8}	1.0013×10^{-7}
$e^{\sin(x)}$	1.4337×10^{-6}	1.4078×10^{-8}

Supplementary Table 14: Average relative errors for the viscous Burgers equation using the frozen at $t = 0$ ($r = 91$) and the time-sampled ($r = 115$) set of custom basis functions. Average error is computed for $t \in [0, 10]$.

$u_0(x)$	Frozen at $t = 0$	Time-sampled
Random	1.8670×10^{-5}	4.2074×10^{-6}
$\sin(x)$	8.1135×10^{-5}	6.8911×10^{-5}
$e^{\sin(x)}$	1.5309×10^{-4}	1.3923×10^{-4}

Supplementary Table 15: Average relative errors for the Korteweg–de Vries equation using the frozen at $t = 0$ ($r = 106$) and the time-sampled ($r = 124$) set of custom basis functions. Average error is computed for $t \in [0, 10]$.

$u_0(x)$	Frozen at $t = 0$	Time-sampled
Random	7.6522×10^{-4}	8.2453×10^{-4}
$\sin(x)$	6.1557×10^{-5}	2.1650×10^{-3}
$e^{\sin(x)}$	4.9445×10^{-4}	5.8131×10^{-4}

Supplementary Table 16: Average relative errors for the Kuramoto–Sivashinsky equation using the frozen at $t = 0$ ($r = 105$) and the time-sampled ($r = 126$) set of custom basis functions. Average error is computed for $t \in [0, 10]$.

$u_0(x)$	Frozen at $t = 0$	Time-sampled
Random	1.7019×10^0	1.6561×10^0
$\sin(x)$	1.8628×10^0	2.1979×10^0
$e^{\sin(x)}$	3.6139×10^{-1}	3.6777×10^{-1}

Supplementary Table 17: Average relative errors for the inviscid Burgers equation using the frozen at $t = 0$ ($r = 128$) and the time-sampled ($r = 312$) set of custom basis functions. Average error is computed for $t \in [0, 10]$.

Representative results for the advection, advection-diffusion, viscous Burgers, Korteweg–de Vries, Kuramoto–Sivashinsky, and inviscid Burgers equations are tabulated in Supplementary Tables 12, 13, 14, 15, 16, and 17, respectively. For each model, results are shown for the in-distribution and out-of-distribution initial conditions. Additionally, a third initial condition, $u(0, x) = e^{\sin(x)}$, similar to that shown in Section 3, is also presented. For all of these results, a sampling gap $\Delta t = 0.05$ for $t \in [0, 1]$ was used in conjunction with a singular value threshold of 10^{-13} for all examples except the inviscid Burgers equation, which used a threshold of 10^{-10}. For the advection-diffusion and Korteweg–de Vries equations, the use of time-sampled basis functions leads to an increase in accuracy relative to using basis functions taken from $t = 0$ for all three initial conditions. For the viscous Burgers equation, the use of time-sampled basis functions leads to a decrease in accuracy relative to using basis functions taken from $t = 0$ for the in-distribution and out-of-distribution initial condition $u(0, x) = \sin(x)$, but an increase in relative accuracy for the initial condition $u(0, x) = e^{\sin(x)}$. For the Kuramoto–Sivashinsky equation, the use of time-sampled basis functions leads to a decrease in the accuracy for all three test initial conditions. The use of time-sampled basis functions for the inviscid Burgers equation leads to a slight increase in accuracy for the in-distribution initial condition. For both the out-of-distribution initial condition and the initial condition $u(0, x) = e^{\sin(x)}$, we find a slight decrease in accuracy. Sampling the trunk function at various times throughout the temporal training domain is the subject of continued investigation and will appear in a future publication.

8 Use the custom-made basis functions from one PDE to expand the solution of another PDE

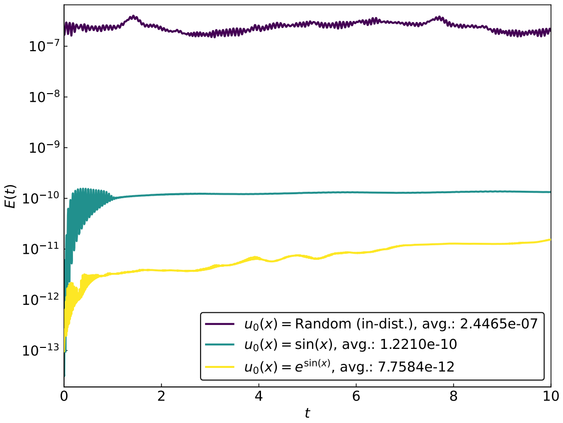

Supplementary Figure 30: Relative errors for the advection equation for $t \in [0, 10]$ and using the custom basis functions found for the viscous Burgers equation ($r = 91$).

For each example PDE, a DeepONet was trained for that specific PDE. However, the natural question arises: can the custom basis functions identified for PDEs that exhibit more complex dynamics be utilized for simpler systems? Supplementary Figure 30 shows results for the advection equation and for the two test initial conditions when using the custom basis functions identified for the viscous Burgers equation ($\nu = 0.1$). From the test case shown in Supplementary Figure 30, we find a slight decrease in the average error for the in-distribution initial condition and the initial condition $u(0, x) = e^{\sin(x)}$, but an increase in the average error for the out-of-distribution initial condition $u(0, x) = \sin(x)$. The concept of using basis functions trained for more complex PDEs for a general class of PDEs is also the subject of continued investigation and will appear in a future publication.

9 Enforcing the periodic boundary conditions during training through a feature expansion

For all the PDEs presented, a discontinuous Galkerin method was utilized to enforce the boundary conditions and obtain a system of ODEs for the expansion coefficients. If a feature expansion [20] is utilized during training, the resulting custom basis functions will individually satisfy the periodic boundary conditions. To enforce periodic boundary conditions during training, a feature expansion utilizing two Fourier basis functions ($x \mapsto \{\sin(x), \cos(x)\}$) was applied to the spatial component of the trunk network input. The ground truth was generated as outlined in Supplementary Section 1 and all other network parameters match those presented in Supplementary Table 1. The mean testing error and corresponding standard deviation based on three training runs are presented in Supplementary Table 18.

PDE	Mean	Standard deviation
Advection	3.36×10^{-5}	3.36×10^{-6}

Supplementary Table 18: DeepONet mean testing errors and standard deviation when using a feature expansion for the advection equation based on three training runs.

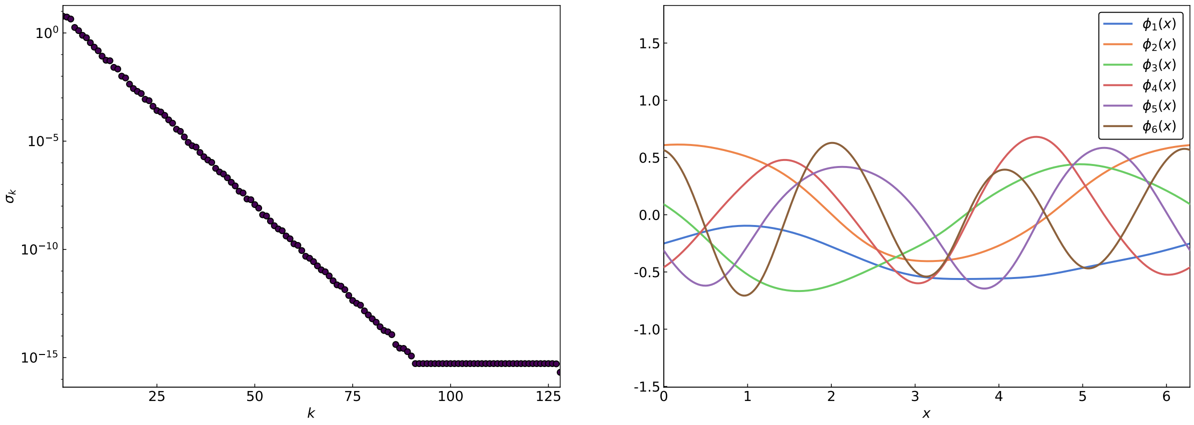

(a) The singular values

(b) The custom basis functions

Supplementary Figure 31: Singular values and the first six custom basis functions for the periodic advection equation when using a feature expansion.

The custom basis functions were constructed following a procedure similar to that outlined in the Methods section, except the quadrature points corresponding to the trapezoid rule and the real trigonometric polynomials were utilized in place of the Gauss-Legendre quadrature points and the orthonormal Legendre polynomials. The $\{\phi_l\}$ can then be evaluated at points away from the

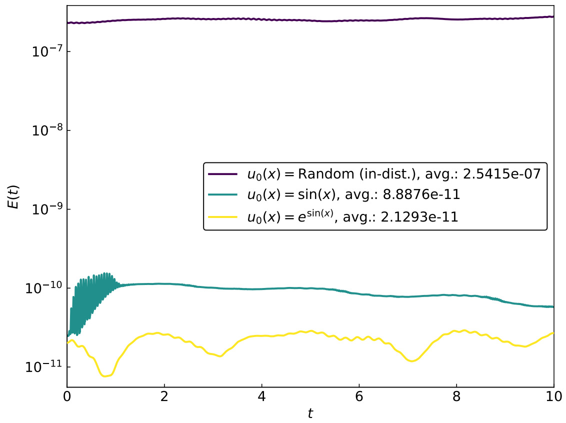

Supplementary Figure 32: Relative errors for the advection equation for $t \in [0, 10]$ when using a feature expansion.

quadrature grid using

$$\tilde{\phi}_l = \frac{1}{2\pi}\sum_{j=0}^{N-1}\phi_l(x_j)w_j + \sum_{k=1}^{N/2-1}\left[\left(\frac{1}{\pi}\sum_{j=0}^{N-1}\phi_l(x_j)\cos(kx_j)w_j\right)\cos(kx) + \left(\frac{1}{\pi}\sum_{j=0}^{N-1}\phi_l(x_j)\sin(kx_j)w_j\right)\sin(kx)\right], \quad \text{for } 1 \le l \le p, \tag{68}$$

where p is the number of trunk net functions evaluated at $t = 0$. If we again drop the tildes on $\{\tilde{\phi}_l\}$ and employ a Galerkin approach (see, e.g., [21, 1] for a detailed discussion) for the advection equation, we obtain a system of ODEs for the expansion coefficients

$$\frac{da_m(t)}{dt} = -\sum_{l=1}^{r} a_l(t)\langle\phi_m, \phi_l'\rangle, \quad m = 1, 2, \ldots, r. \tag{69}$$

A singular value threshold of 10^{-10} ($r = 61$), 2^7 quadrature points and auto-differentiation were utilized. The numerical integrator and associated tolerances were specified to match those used for the discontinuous Galerkin approach. The singular value spectrum and the first six custom basis functions are shown in Supplementary Figure 31. The relative errors compared to the $M = 128$ Fourier solution are presented in Supplementary Figure 32 for the same in-distribution and out-of-distribution initial conditions shown in supplement Section 1. From the test cases shown in Supplementary Figure 32, we find a slight decrease in the error for the in-distribution initial condition and the initial condition $u(0, x) = e^{\sin(x)}$, while for the out-of-distribution initial condition $u(0, x) = \sin(x)$, we find a slight increase in the error. In addition to using the real trigonometric polynomials to evaluate the $\{\phi_l\}$ at locations away from the quadrature grid, the use of B-Splines are also being investigated. The concepts of using a feature expansion and hard constraints to enforce boundary conditions during training are the subject of continued investigation and will appear in a future publication.

10 Results for the Dirichlet problem when trained using initial conditions sampled from a non-periodic Gaussian random field

For all the results presented, the initial conditions utilized for training the DeepONets that underlie our construction were sampled from a periodic Gaussian random field as outlined in Supplementary Section 1. In this section, results are presented for the advection-diffusion equation ($\alpha = 1.0$, $\nu = 0.1$) with Dirichlet boundary conditions where the underlying DeepONet was trained using initial conditions sampled from a non-periodic Gaussian random field.

To generate the initial conditions for training the DeepONets, $f(x)$ was sampled from a mean zero Gaussian random field with covariance kernel given by Supplementary Equation (1). Twenty-five example non-periodic initial conditions are shown in Supplementary Figure 33. The ground truth data was generated by writing the solution in terms of $L = 127$ orthonormal Legendre polynomials (see Supplementary Equation (3)) and with the boundary conditions enforced using a discontinuous Galerkin approach as outlined in Supplementary Section 4. The Dirichlet boundary conditions were specified to be the values of the initial condition at the endpoints $x = 0$ and $x = 2\pi$ for all time. A 128-node Gauss–Legendre quadrature grid along with an explicit, singly diagonal, implicit Runge–Kutta integrator with adaptive step size, relative error tolerance 10^{-10}, and absolute error tolerance 10^{-14} was utilized to generate the ground truth and evolve the custom basis function solutions. The solution was saved at time values of 10^{-3} apart. All other DeepONet training parameters match those presented in Supplementary Table 1. The mean testing error and corresponding standard deviation based on three training runs are presented in Supplementary Table 19.

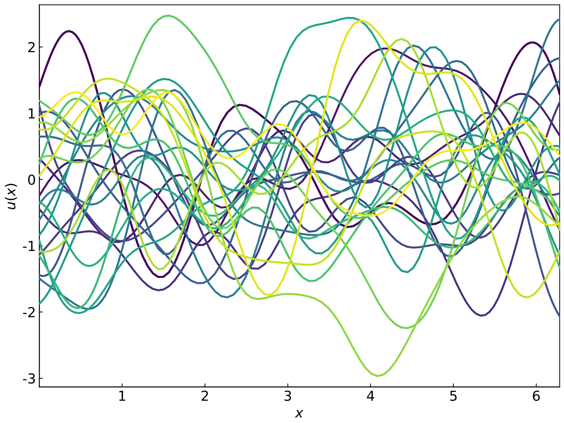

Supplementary Figure 33: Twenty-five example non-periodic initial conditions sampled from the Gaussian random field.

PDE	Mean	Standard deviation
Advection-diffusion	5.16×10^{-4}	2.36×10^{-4}

Supplementary Table 19: DeepONet mean testing errors and standard deviation for the advection-diffusion equation with Dirichlet boundary conditions and trained using non-periodic initial conditions based on three training runs.

The singular value spectrum and the first six custom basis functions are shown in Supplementary

Figure 34, while the random in-distribution initial condition is presented in Supplementary Figure 35. In addition to the random in-distribution initial condition, the initial conditions $u_0(x) = \sin(x)$ and $u_0(x) = e^{\sin(x)}$ were also evaluated. Supplementary Figure 36 shows the evolution of the relative errors for the temporal domain $t \in [0, 10]$ when using $r = 99$ custom basis functions. From Supplementary Figure 36, we find good agreement for all three test initial conditions. The spatiotemporal plots for the three test initial conditions are shown in Supplementary Figures 37, 38, and 39. The mean wall-clock times for the $r = 99$ custom basis function, $L = 98$ Legendre, and $L = 127$ Legendre solutions are shown in Supplementary Table 20.

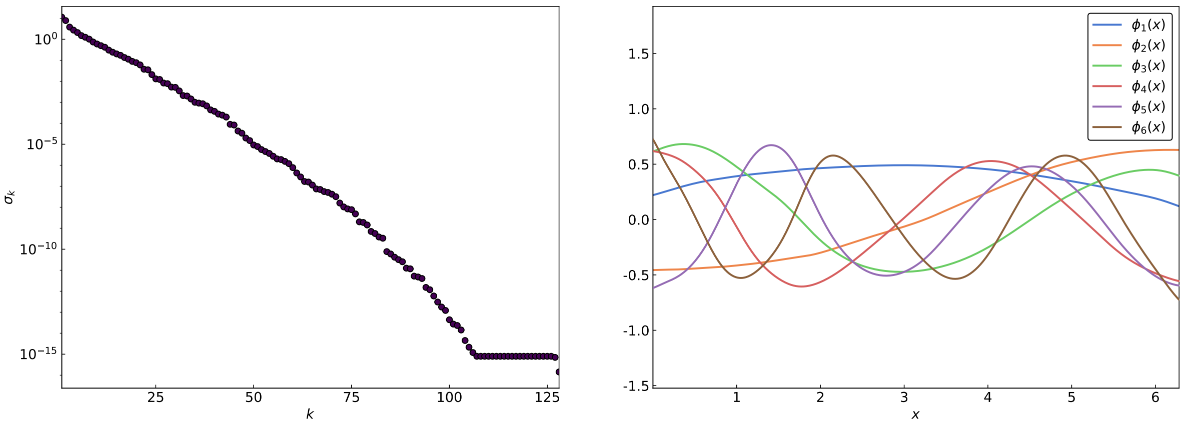

(a) Singular value spectrum

(b) The custom basis functions

Supplementary Figure 34: Singular value spectrum and first six custom basis functions for the Dirichlet advection-diffusion problem trained using non-periodic initial conditions.

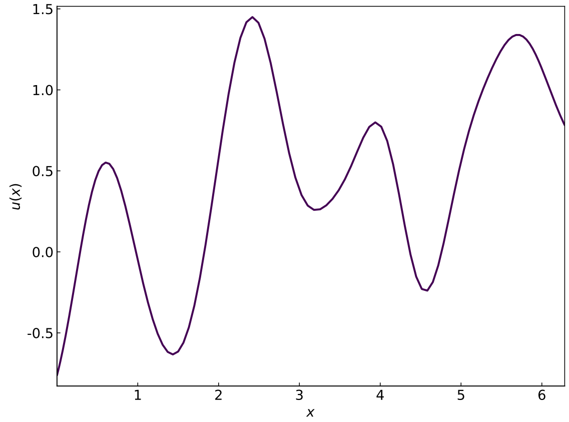

Supplementary Figure 35: In-distribution initial condition for the Dirichlet advection-diffusion problem trained using non-periodic initial conditions.

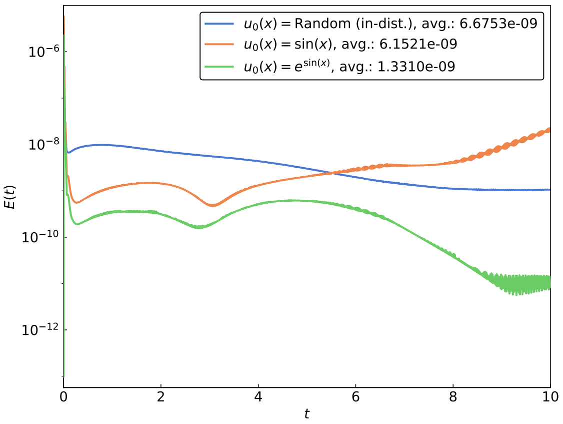

Supplementary Figure 36: Relative errors for the Dirichlet advection-diffusion problem trained using non-periodic initial conditions for $t \in [0, 10]$.

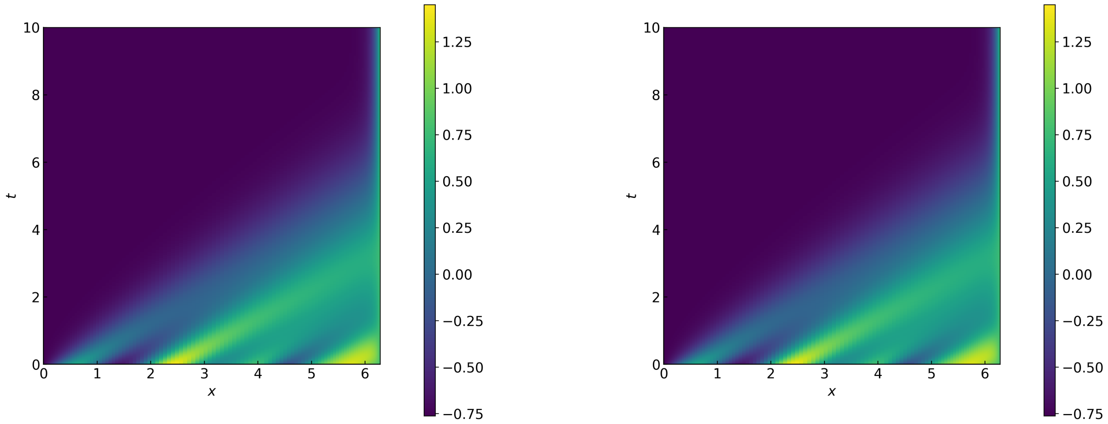

(a) Legendre solution

(b) Custom basis solution

Supplementary Figure 37: Spatiotemporal plots for the in-distribution initial condition for the Dirichlet advection-diffusion problem trained using non-periodic initial conditions.

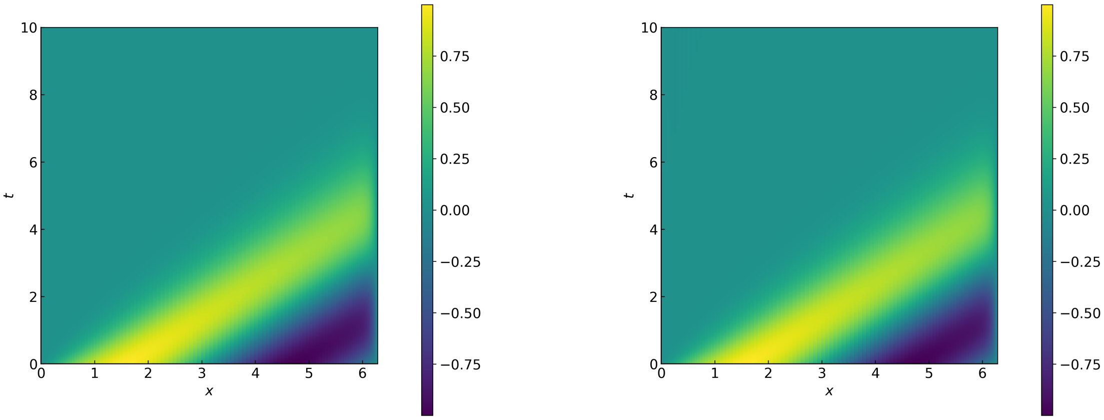

(a) Legendre solution (b) Custom basis solution

Supplementary Figure 38: Spatiotemporal plots for the initial condition $u_0(x) = \sin(x)$ for the Dirichlet advection-diffusion problem trained using non-periodic initial conditions.

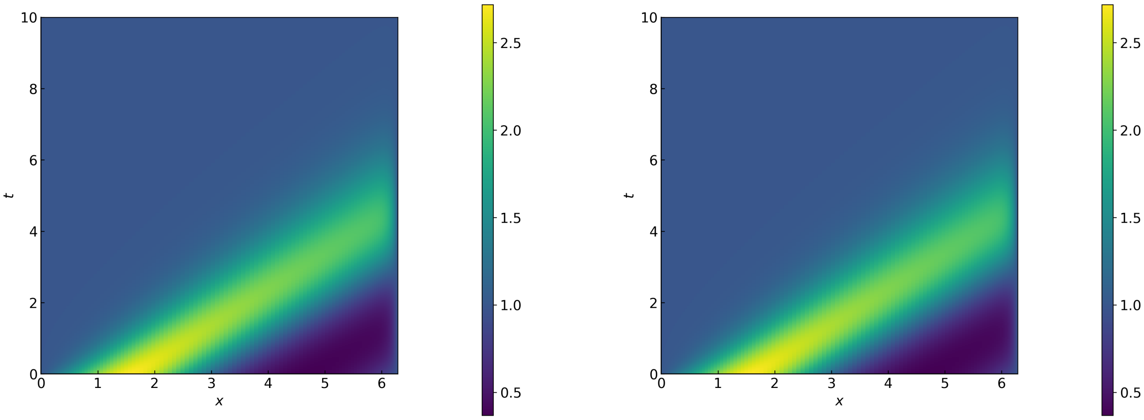

(a) Legendre solution (b) Custom basis solution

Supplementary Figure 39: Spatiotemporal plots for the initial condition $u_0(x) = e^{\sin(x)}$ for the Dirichlet advection-diffusion problem trained using non-periodic initial conditions.

Solution method	Mean wall-clock time (s)	Average relative error
Custom basis function solution ($r = 99$)	1.310812	6.6753×10^{-9}
Legendre solution ($L = 98$)	1.467361	9.5663×10^{-11}
Legendre reference solution ($L = 127$)	2.008042	–

Supplementary Table 20: Mean wall-clock times in seconds and average relative errors for the advection-diffusion equation with Dirichlet boundary conditions computed using the custom basis functions developed for non-periodic initial conditions and Legendre solution methods. The average errors were computed for $t \in [0, 10]$.

References

[1] Claudio Canuto, M Yousuff Hussaini, Alfio Quarteroni, A Thomas Jr, et al. *Spectral methods in fluid dynamics*. Springer Science & Business Media, 2012.

[2] Christopher Rackauckas and Qing Nie. Differentialequations.jl–a performant and feature-rich ecosystem for solving differential equations in julia. *Journal of Open Research Software*, 5(1), 2017.

[3] Richard H Pletcher, John C Tannehill, and Dale Anderson. *Computational fluid mechanics and heat transfer*. CRC press, 2012.

[4] HQ Yang and AJ Przekwas. A comparative study of advanced shock-capturing schemes applied to Burgers' equation. *Journal of Computational Physics*, 102(1):139–159, 1992.

[5] Kookjin Lee, Nathaniel A Trask, Ravi G Patel, Mamikon A Gulian, and Eric C Cyr. Partition of unity networks: deep hp-approximation. *arXiv preprint arXiv:2101.11256*, 2021.

[6] Nat Trask, Mamikon Gulian, Andy Huang, and Kookjin Lee. Probabilistic partition of unity networks: clustering based deep approximation. *arXiv preprint arXiv:2107.03066*, 2021.

[7] Hassler Whitney. Analytic extensions of differentiable functions defined in closed sets. *Transactions of the American Mathematical Society*, 36(1):63–89, 1934.

[8] Lars Hörmander. *The analysis of linear partial differential operators I: Distribution theory and Fourier analysis*. Springer, 2015.

[9] John P Boyd. A comparison of numerical algorithms for Fourier extension of the first, second, and third kinds. *Journal of Computational Physics*, 178(1):118–160, 2002.

[10] Oscar P Bruno and Mark Lyon. High-order unconditionally stable FC-AD solvers for general smooth domains I. Basic elements. *Journal of Computational Physics*, 229(6):2009–2033, 2010.

[11] Ben Adcock and Daan Huybrechs. Approximating smooth, multivariate functions on irregular domains. In *Forum of Mathematics, Sigma*, volume 8. Cambridge University Press, 2020.

[12] Saad Qadeer and Boyce E Griffith. The smooth forcing extension method: A high-order technique for solving elliptic equations on complex domains. *Journal of Computational Physics*, 439:110390, 2021.

[13] Beichuan Deng, Yeonjong Shin, Lu Lu, Zhongqiang Zhang, and George Em Karniadakis. Convergence rate of deeponets for learning operators arising from advection-diffusion equations. *arXiv preprint arXiv:2102.10621*, 2021.

[14] Samuel Lanthaler, Siddhartha Mishra, and George Em Karniadakis. Error estimates for deeponets: A deep learning framework in infinite dimensions. *arXiv preprint arXiv:2102.09618*, 2021.

[15] Chi-Wang Shu et al. Different formulations of the discontinuous Galerkin method for the viscous terms. *Advances in Scientific Computing*, pages 144–155, 2001.

[16] Bernardo Cockburn and Chi-Wang Shu. The local discontinuous galerkin method for time-dependent convection-diffusion systems. *SIAM Journal on Numerical Analysis*, 35(6):2440–2463, 1998.

[17] Chi-Wang Shu. Discontinuous Galerkin methods: general approach and stability. *Numerical solutions of partial differential equations*, 201, 2009.

[18] Jue Yan and Chi-Wang Shu. A local discontinuous Galerkin method for KdV type equations. *SIAM Journal on Numerical Analysis*, 40(2):769–791, 2002.

[19] Yan Xu and Chi-Wang Shu. Local discontinuous Galerkin methods for the Kuramoto–Sivashinsky equations and the Ito-type coupled KdV equations. *Computer methods in applied mechanics and engineering*, 195(25-28):3430–3447, 2006.

[20] Lu Lu, Xuhui Meng, Shengze Cai, Zhiping Mao, Somdatta Goswami, Zhongqiang Zhang, and George Em Karniadakis. A comprehensive and fair comparison of two neural operators (with practical extensions) based on fair data. *arXiv preprint arXiv:2111.05512*, 2021.

[21] Boyd, J. P. *Chebyshev and Fourier Spectral Methods.* Dover, 2001.
